# Clinical Significance of Pre-treated Neutrophil-Lymphocyte Ratio in the Management of Urothelial Carcinoma: A Systemic Review and Meta-Analysis

**DOI:** 10.3389/fonc.2019.01365

**Published:** 2019-12-16

**Authors:** Jungyo Suh, Jae Hyun Jung, Chang Wook Jeong, Cheol Kwak, Hyeon Hoe Kim, Ja Hyeon Ku

**Affiliations:** ^1^Department of Urology, Seoul National University College of Medicine, Seoul, South Korea; ^2^Department of Urology, Seoul Metropolitan Government- Seoul National University Boramae Medical Center, Seoul, South Korea

**Keywords:** urothelial carcinoma, neutrophil-lymphocyte ratio, trans-urethral resection of bladder tumor, cystectomy, chemotherapy, oncological outcome

## Abstract

**Purpose:** We performed a study-level meta-analysis to summarize the current evidence on the correlation between pretreatment neutrophil-to-lymphocyte ratios (NLR) and oncological outcomes in each type of management for urothelial carcinoma.

**Method:** All articles published until February 2017 in PubMed, Scopus, and EMBASE database were collected and reviewed. The current evidence on correlations between pretreatment NLR and oncological outcomes in each type of management for urothelial carcinoma, including transurethral resection of bladder tumor (TURBT), radical cystectomy (RCx), chemotherapy (CTx), and nephroureterectomy (NUx), were summarized.

**Results:** Thirty-eight studies containing clinical information on 16,379 patients were analyzed in this study. Pooled hazard ratios (HR) and odds ratios (OR) with 95% confidence intervals were calculated after weighing each study. Heterogeneity among the studies and publication bias were assessed. Pretreatment NLR was significantly associated with muscle invasiveness (OR: 4.27), recurrence free survival (RFS, HR: 2.32), and progression-free survival (PFS, HR: 2.45) in TURBT patients. In the RCx patients, high NLR was negatively associated with both disease status (extravesical extension and lymph-node positivity, OR: 1.14 and 1.43, respectively) and oncological outcomes [overall survival (OS), PFS], and cancer specific survival (CSS, HR: 1.18, 1.12, and 1.35, respectively). Pretreatment NLR was negatively correlated with pathologic downstaging (OR: 0.79) and positively correlated with PFS (HR: 1.30) and OS (HR: 1.44) in CTx patients. For patients who underwent NUx, pretreatment NLR was significantly associated with OS (HR: 1.72), PFS (HR: 1.63), and CSS (HR: 1.68).

**Conclusions:** Pretreatment NLR is a useful biomarker for disease aggressiveness, oncological outcome, and treatment response in the management of patients with urothelial carcinoma. More evidence is needed to clarify these results.

## Introduction

Urothelial carcinoma of the bladder is the third most common and the eighth most lethal malignancy in the United States (1), showing a high incidence in developed countries (2). This malignancy originates from normal urothelial cells and can occur in any part of the urinary tract, including the renal pelvis, ureter, bladder, and urethra. The majority of urothelial carcinomas is present in the lower urinary tract, mostly in the bladder. Muscle invasiveness is the most important parameter in the management of bladder urothelial carcinoma. Non-muscle invasive bladder cancer (NMIBC) can be managed by transurethral resection of bladder tumors (TURBT). Muscle-invasive bladder cancer (MIBC) is treated with radical surgery or systemic treatment. The standard treatment for upper urinary tract urothelial carcinoma (UTUC) is radical nephroureterectomy with bladder cuffing. Since urothelial carcinoma shows a variety of clinical manifestations, selecting the proper treatment is one of the most critical issues in the management of patients with urothelial carcinoma.

Recent studies have revealed that the inflammatory response plays an essential role in tumor development, progression, and prognosis (3). Elevation of C-reactive protein (CRP), the presence of some cytokines, and changes in the proportion of white blood cells in the peripheral blood are common findings reflecting the systemic inflammatory response. Among these changes, the peripheral neutrophil-to-lymphocyte ratio (NLR) is one of the most widely studied prognostic biomarkers in many solid tumors because of its easy calculation and cost-effectiveness (4). High NLR tends to be negatively correlated with poor survival in urothelial carcinoma (5). Some studies have suggested a link between high NLR with the pathologic stage of urothelial carcinoma, including muscle invasiveness (6, 7), extravesical extension (8–10), and lymph-node positivity (10, 11). Moreover, recent research has indicated that NLR is a predictive biomarker of treatment response (12). However, one large-scale study did not find a correlation between high preoperative NLRs and extra-vesical extension (11). Thus, the clinical utility of preoperative NLR as a prognostic or predictive biomarker in urothelial carcinoma is still controversial.

Therefore, the objective of this study was to determine the correlation between pretreatment NLRs and oncological outcomes in each type of management for urothelial carcinoma, including TURBT, radical cystectomy (RCx), chemotherapy (CTx), and nephroureterectomy (NUx), through a meta-analysis of published studies.

## Materials and Methods

### Search Strategy

This study was performed following the Preferred Reporting Items for Systematic Reviews and Meta-Analyses (PRISMA) guidelines. The PubMed, SCOPUS, and EMBASE databases were searched to collect suitable literature published up to February 2017. The search used a combination of “Neutrophil; Lymphocyte; Bladder; Cancer” and “Neutrophil; Lymphocyte; Urothelial; and Cancer” terms. To identify additional shrouded studies, we carefully examined the references of each article.

### Inclusion and Exclusion Criteria

Two reviewers (CWJ and CK) screened all titles and abstracts of the initially searched articles. After screening, the full-text articles were separately evaluated by two different reviewers (HHK and JHK) to determine study eligibility. Disagreement on study eligibility was resolved through discussion. The inclusion criteria for eligibility were that the report: (1) investigated urothelial carcinoma, (2) had patient neutrophil-to-lymphocyte ratios, (3) evaluated the relationship between pathologic features and prognosis, and (4) had enough information to calculate odds ratios (OR), or hazard ratios (HR) with 95% confidence intervals (CI). Studies not written in English, case reports, editorial letters or reviews, and those not performed in humans were excluded. If the investigations were conducted on similar patients by the same research group, only the largest and newest article was included in the systematic review.

### Data Extraction and Handling

Two reviewers (JHJ and JS) independently extracted information from the selected studies. Data tables were constructed to record all associated data from the texts, tables, and figures of each study. The study information (name of authors, publication year, region), the number of patients, follow-up information, and disease status were obtained. After completing the data tables for each study, both reviewers compared their results and arrived at a consensus for any differences.

### Statistical Analysis

The DerSimonian and Laird random-effects model (13) was selected to weigh each study for the meta-analysis. Odds ratios and 95% CIs were used to assess the relationship between NLR and disease status for each specific situation, including muscle invasion in TUR, extravesical extension, or lymph node positivity in radical cystectomy, and pathologic downstaging after neoadjuvant chemotherapy. Hazard ratios and 95% CI were used to estimate the effects of NLR on survival. The heterogeneity of the studies was assessed by Chi-squared tests. Statistical significance was considered for *p*-values of < 0.05. The *I*^2^ statistic was also calculated to determine the heterogeneity of the studies. *I*^2^ values larger than 75%, <25%, and between 25 and 75% indicated a high risk of heterogeneity among studies, no heterogeneity, and moderate heterogeneity among studies, respectively.

Publication bias was assessed by funnel plots, rank correlation analysis (Begg test), and linear regression analysis (Egger test). In the funnel plots, a symmetric, inverted funnel shape indicated no publication bias. A *p*-value < 0.05 by Begg and Egger's test was considered publication bias (Supplementary Data 1). The pooled ORs and HRs of the meta-analyses were calculated using RevMan 5.0 (the Cochrane Collaboration, Copenhagen) software. All statistical analyses were performed with R program 3.5.0 (R Development Core Team, Vienna, http://www.R-project.org).

**Figure 1:**
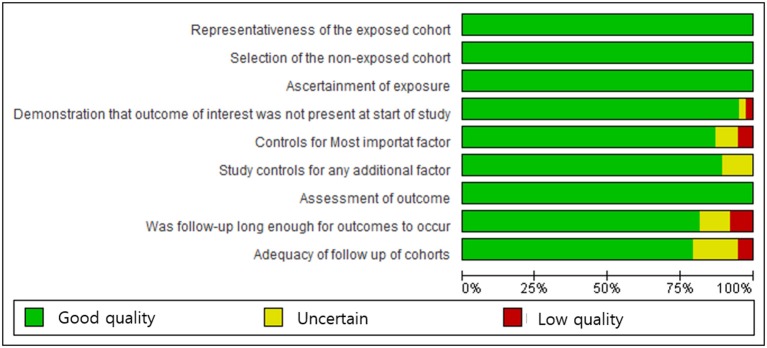
Newcastle-Ottawa Scale graph: the review authors' judgments on each parameter are presented as percentages across all included studies.

Data quality and the risk of bias assessment were performed by three investigators (JHK, JS, and JHJ). Each reviewer independently read the published articles and performed a quality assessment based the Newcastle-Ottawa Scale (NOS) (14). The NOS assesses the methodologic quality of each study in three domains: selection of the study groups, comparability of the groups, and ascertainment of exposure and outcome. The risk of bias was stratified on three levels. Quality scores of NOS of >7, 4 to 6, and < 4 indicated high quality, moderate quality, and low-quality studies, respectively. The methodologic quality scores of all included studies are shown in Figure 1. Specific quality assessment scores and data are shown in Supplementary Datas 2, 3.

## Results

### Literature Search and Study Selection

**Figure 2:**
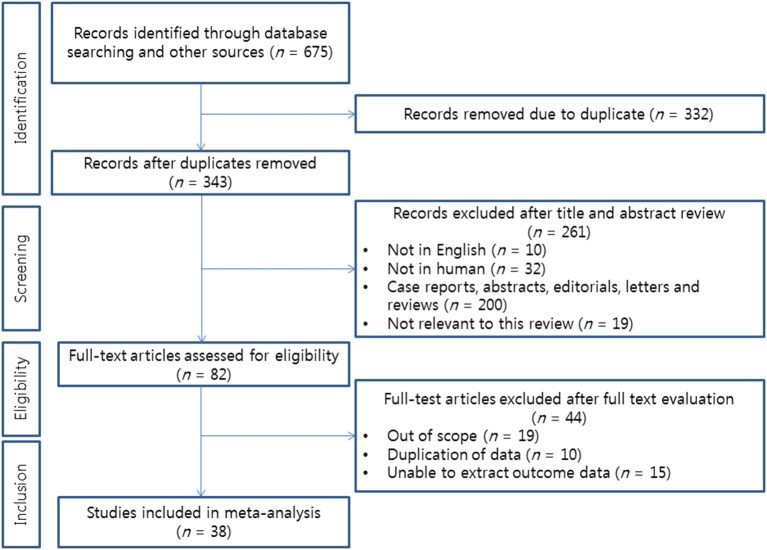
Flow chart of the literature search for this meta-analysis.

A total of 675 articles was primarily identified by database searching. After removing duplicated work, 343 articles remained for screening. After reviewing the running title and abstract by two investigators, 82 articles remained eligible for assessment. Full-text reviews were performed by the two investigators. Finally, 38 studies were selected for meta-analysis (Figure 2), including seven articles on TURBT (6, 7, 15–19), 15 on radical cystectomy (8–12, 20–28), five on chemotherapy (29–33), and 11 on nephroureterectomy for upper ureter urothelial carcinoma (34–44).

### Study Characteristics

The characteristics of the selected 38 studies are summarized in Tables 1–4. Seven articles evaluated the clinical significance of NLR in TURBT patients. Only two studies were eligible for assessing the link between NLR and invasiveness in TURBT patients (6, 7). One study performed in Italy was prospectively designed (16). Others were retrospective studies. The majority of those studies were conducted in Turkey (7, 17, 19). However, a study from Japan had the largest study population (15). The cutoff value for NLR varied from 2.2 to 3.89 depending on the study (Table 1). Fifteen articles were eligible for a meta-analysis of radical cystectomies (Table 2). Only extravesical extension, lymph node positivity, and downstaging after NAC had sufficient information to evaluate the correlation between preoperative NLR and disease status. Regarding the parameters of oncological outcomes, cancer-specific survival (CSS), overall survival, and progression-free survival (PRS) had sufficient data for linkage analysis with preoperative NLRs. Most studies set cutoff values for NLR from 2.3 to 5, however, three studies used it as a continuous variable (12, 23, 25). The studies were conducted in many countries. Two large multi-center retrospective studies were performed in the USA (10) and Europe (11). Five articles were eligible for correlation analysis of pretreatment NLRs with oncologic outcomes in chemotherapy patients (Table 3). All studies provided information for overall survival. However, only two studies (31, 32) provided progression-free survival data. European, American, and East Asian (Japan, China) data were included in this meta-analysis. The cutoff values for the NLR ranged from 3 to 5. One study conducted a retrospective analysis from 10 prospective phase II clinical trials (29). Eleven studies were eligible for meta-analysis of NLR association with oncological outcomes in nephroureterectomy patients (Table 4). Eight studies provided enough information to analyze the relationship between preoperative NLRs and PFS (34, 36, 39–42, 44, 46) and CSS (34, 36, 39–42, 44, 46). Most of those studies were conducted in East Asia. Two studies contained information on Western population patients (34, 43). All studies used NLR as a discrete variable, with cutoff values ranging from 2.0 to 3.22.

**Table 1:** Characteristics of studies eligible for TURBT analysis.

**Study**	**Country**	**Publication year**	**Recruitment period**	**Number patients**	**Study design**	**Inclusion and exclusion criteria**	**NLR cutoff value**	**Eligible for correlation analysis with clinicopathologic features**	**Eligible for correlation analysis with oncological outcomes**	**The Newcastle-Ottawa Scale**	**Quality of study**
Lee et al. (6)	Korea	2015	2011–2013	226	Retrospective	Yes	3.89	Invasiveness	No	7	High
Can et al. (7)	Turkey	2012	2001–2011	182	Retrospective	Yes	2.57	Invasiveness	No	6	Moderate
Ozyalvacli et al. (17)	Turkey	2015	2008–2013	166	Retrospective	Yes	2.43	No	RFS, PFS	7	High
Mano et al. (18)	Israel	2015	2003–2010	91	Retrospective	Yes	2.41	No	RFS, PFS	7	High
Favilla et al. (16)	Italia	2016	2008–2014	178	Prospective	Yes	3	No	RFS, PFS	9	High
Ogihara et al. (15)	Japan	2016	1995–2013	605	Retrospective	Yes	2.2	No	RFS, PFS	9	High
Camtosun et al. (19)	Turkey	2017	2007–2014	89	Retrospective	Yes	2.5	No	RFS	5	Moderate

*NLR, neutrophil to lymphocyte ratio; TURBT, transurethral resection of bladder tumor; RFS, recurrence-free survival; PFS, progression-free survival*.

**Table 2:** Characteristics of studies eligible for radical cystectomy analysis.

**Study**	**Country**	**Publication year**	**Recruitment period**	**Number of patients**	**Study design**	**Inclusion and exclusion criteria**	**NLR cutoff value**	**Eligible for correlation analysis with pathologic status**	**Eligible for correlation analysis with oncological outcomes**	**The Newcastle-Ottawa Scale**	**Quality of study**
Krane et al. (8)	USA	2013	2005–2011	68	Retrospective	Yes	2.5	Extravesical invasion	CSS, OS	8	High
Potretzke et al. (9)	USA	2014	2002–2012	102	Retrospective	Yes	Median 4.33^*^	Extravesical invasion	No	9	High
Viers et al. (10)	USA	2014	1994–2005	899	Retrospective	Yes	2.7	Extravesical invasion, LN positivity	CSS, OS	9	High
D'Andrea et al. (11)	Europe	2017	1990–2012	4198	Retrospective	Yes	3.5	Extravesical invasion, LN positivity	CSS, OS, PFS	9	High
Seah et al. (20)	Canada	2015	2006–2013	26	Retrospective	Yes	Median 2.3^†^	Downstaging after NAC	No	9	High
Buisan et al. (25)	Spain	2017	2007–2015	75	Retrospective	Yes	As continuous variable	Downstaging after NAC	No	9	High
Nguyen et al. (23)	USA	2016	2001–2015	310	Retrospective	Yes	As continuous variable	No	CSS, OS, PFS	7	High
Morizawa et al. (26)	Japan	2016	2002–2013	110	Retrospective	Yes	2.6	No	CSS, OS, PFS	8	High
Bhindi et al. (27)	Canada	2016	1992–2012	418	Retrospective	Yes	Median 2.9	No	CSS, OS, PFS	8	High
Ku et al. (21)	Korea	2015	1999–2011	419	Retrospective	Yes	5	No	CSS, OS	9	High
Yoshida et al. (24)	Japan	2016	1995–2014	323	Retrospective	Yes	2.7	No	OS	9	High
Ojerholm et al. (12)	USA	2017	1987–1998	230	Prospective cohort^‡^	Yes	As continuous variable	No	OS	8	High
Kawahara et al. (45)	Japan	2016	1999–2014	74	Retrospective	Yes	2.38	No	OS	7	High
Gondo et al. (22)	Japan	2012	2000–2009	189	Retrospective	Yes	2.5	No	CSS	8	High
Ozcan et al. (28)	Turkey	2015	1990–2013	363	Retrospective	Yes	2.5	No	CSS	7	High

* *Calculated cutoff value for upstage to non-organ confined disease in this study*.

† *Median pre-NAC NLR value was used for this meta-analysis*.

‡ *The study was a secondary planned analysis from a SWOG-8710 prospective cohort*.

*NLR, neutrophil to lymphocyte ratio; CSS, cancer-specific survival; OS, overall survival; PFS, progression-free survival; NAC, neoadjuvant chemotherapy*.

**Table 3:** Characteristics of studies eligible for chemotherapy analysis.

**Study**	**Country**	**Publication year**	**Recruitment period**	**Number of patients**	**Study design**	**Inclusion and exclusion criteria**	**NLR cutoff value**	**Eligible for correlation analysis with oncological outcomes**	**The Newcastle-Ottawa Scale**	**Quality of study**
Taguchi et al. (30)	Japan	2015	2003–2011	200	Retrospective	Yes	3	OS	9	High
Rossi et al. (31)	Europe	2015	2003–2012	292	Retrospective	Yes	3	OS, PFS	9	High
Sonpavde et al. (29)	Multi-region (USA, Europe, Canada)	2016	2000–2016	708	Retrospective^*^	Yes	5	OS	8	High
Auvary et al. (32)	Europe (France, Turkey)	2016	2002–2014	208	Retrospective	Yes	3.2	OS, PFS	9	High
Su et al. (33)	China	2017	1997–2014	256	Retrospective	Yes	3.0	OS	9	High

* *This study retrospectively reviewed 10 phase II prospective trials*.

*NLR, neutrophil to lymphocyte ratio; OS, overall survival; PFS, progression free survival*.

**Table 4:** Characteristics of studies eligible for nephroureterectomy analysis.

**Study**	**Country**	**Publication year**	**Recruitment period**	**Number of patients**	**Study design**	**Inclusion and exclusion criteria**	**NLR cutoff value**	**Eligible for correlation analysis with oncological outcomes**	**The Newcastle-Ottawa Scale**	**Quality of study**
Azuma et al. (44)	Japan	2016	1998–2008	137	Retrospective	Yes	2.5	CSS, PFS	9	High
Tanaka et al. (41)	Japan	2014	1993–2011	665	Retrospective	Yes	3.0	CSS, PFS	9	High
Luo et al. (42)	China	2014	2004–2010	234	Retrospective	Yes	3.0	CSS, PFS	9	High
Kim et al. (39)^†^	Korea	2015	1999–2010	277	Retrospective	Yes	5.0	CSS, PFS	9	High
Sung et al. (40)	Korea	2015	1994–2011	410	Retrospective	Yes	2.5	PFS	9	High
Song et al. (36)	China	2016	2005–2011	140	Retrospective	Yes	2.2	PFS	9	High
Ito et al. (46)	Japan	2016	1999–2013	71	Retrospective	Yes	2.0	PFS	9	High
Vartolomei et al. (34)	Multi-region (USA, Europe, Canada)	2017	1990–2008	2477	Retrospective	Yes	2.7	PFS, CSS	9	High
Dalpiaz et al. (43)	Europe	2014	1990–2012	202	Retrospective	Yes	2.7	CSS, OS	9	High
Huang et al. (35)	China	2016	2002–2013	481	Retrospective	Yes	3.22	CSS, OS	9	High
Cheng et al. (38)	Taiwan	2016	2005–2010	420	Retrospective	Yes	2.7	CSS, OS	9	High

† *This study provided two values for NLR, the first one was the actual NLR from each neutrophil and lymphocyte count. The second one was a derived NLR, which was calculated from: neutrophil count/(white blood cell count – neutrophil count). The two NLR values had different cutoff values. We used the actual NLRs for consistency*.

*NLR, neutrophil to lymphocyte ratio; OS, overall survival; PFS, progression free survival; CSS, cancer specific survival*.

### Clinical Significance of NLR in TURBT Patients

#### Correlation of NLR With Clinicopathologic Feature (Invasiveness)

**Figure 3:**
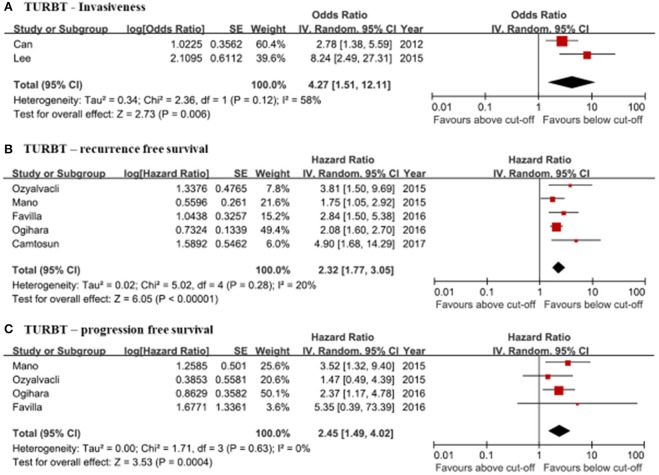
Forrest plots of the relationship between neutrophil-to-lymphocyte ratios (NLR) and clinical information of the TURBT patients. **(A)** TURBT—Invasiveness. **(B)** TURBT—recurrence free survival. **(C)** TURBT—progression free survival.

Only two studies reported the correlation between preoperative NLRs and muscle invasiveness. The pooled OR was 4.27 (95% CI: 1.51–27.31) and moderate level of inter-study heterogeneity was present (*I*^2^ = 58%, *p* = 0.12) (Figure 3A). Publication bias was not assessable owing to the limited number of studies.

#### Correlation of NLR With Oncological Outcomes (Recurrence, Progression)

Five studies were eligible for assessing the relationship between NLRs and RFS (15–19). The results from the meta-analysis of RFS showed a negative association between high NLRs and RFS. The pooled HR was 2.32 (95% CI: 1.77–3.05). Heterogeneity was not present (*I*^2^ = 20%, *p* = 0.28) (Figure 3B). PFS was analyzed using data from four studies (15–18). NLRs above the cutoff value of each study were associated with a higher probability of recurrence (pooled HR: 2.45, 95% CI: 1.49–4.02). Inter-study heterogeneity was not found (*I*^2^ = 0%, *p* = 0.63) (Figure 3C). Publication bias was not founded in either meta-analysis.

### Clinical Significance of NLR in Radical Cystectomy Patients

#### Correlation of NLR With Clinicopathologic Features (Extravesical Extension, Lymph Node Positivity) and Prediction of Clinical Response (Downstaging After Neoadjuvant Chemotherapy)

**Figure 4:**
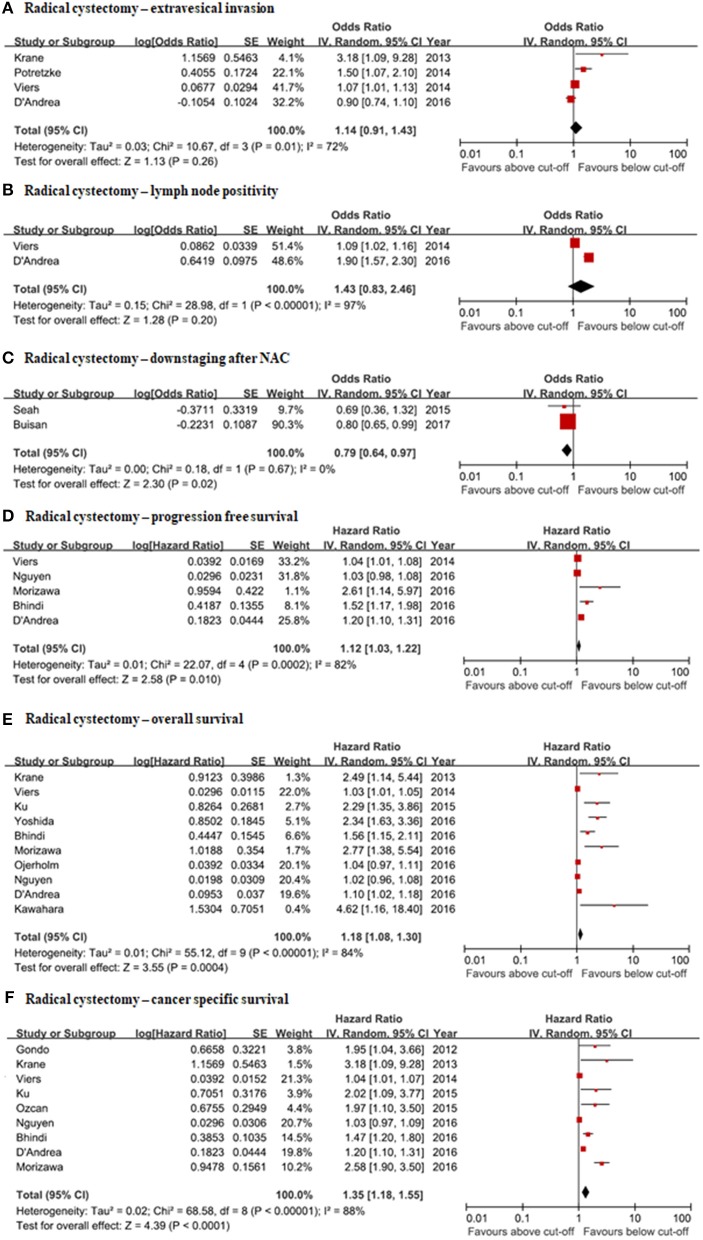
Forrest plots of the relationship between NLRs and clinical information of radical cystectomy patients. **(A)** Radical cystectomy—extravesical invasion. **(B)** Radical cystectomy—lymph node positivity. **(C)** Radical cystectomy—downstaging after NAC. **(D)** Radical cystectomy—progression free survival. **(E)** Radical cystectomy—overall survival. **(F)** Radical cystectomy—cancer specific survival.

In four studies (8–11), extravesical extension was evaluated for pathologic up-staging to analyze the correlation with preoperative NLRs. The pooled OR was 1.14 (95% CI: 0.91–1.43) and inter-study heterogeneity was present (*I*^2^ = 72%, *p* = 0.01) (Figure 4A). The correlation between NLRs and lymph node (LN) positivity was assessed in two large, retrospective studies (10, 11). The pooled OR was 1.43 (95% CI: 0.83–2.46). Heterogeneity between the two studies was highly present (*I*^2^ = 97%, *p* < 0.00001) (Figure 4B). Pathologic downstaging after neoadjuvant chemotherapy was evaluated in two studies (20, 25). The pooled OR was calculated to be 0.79 (95% CI: 0.64–0.99). Heterogeneity between the two studies was not present (*I*^2^ = 0%, *p* = 0.67) (Figure 4C). Publication biases could not be assessed due to the limited number of studies for LN positivity and pathologic downstaging after NAC. Publication bias was not found in the meta-analysis of extravesical extension.

#### Correlation of NLR With Oncological Outcomes (Overall Survival, Cancer-Specific Survival, Progression-Free Survival)

PFS was evaluated by meta-analysis in five studies (10, 11, 23, 26, 27). The pooled hazard ratio was 1.12 (95% CI: 1.03–1.31). There was a high inter-study heterogeneity (*I*^2^ = 82%, *p* = 0.0002) (Figure 4D). Ten studies were eligible for assessing pooled HRs of the NLRs associated with overall survival in radical cystectomy patients (8, 10–12, 21, 23, 24, 26, 27, 45). The pooled HR from meta-analysis was 1.18 (95% CI: 1.08–1.30). There was high inter-study heterogeneity (*I*^2^ = 84%, *p* < 0.00001) (Figure 4E). Nine studies had qualified data for evaluating the correlation of NLR with cancer-specific survival (8, 10, 11, 21–23, 26–28). The pooled HR was 1.35 (95% CI: 1.18–1.55). There was high heterogeneity (*I*^2^ = 88%, *p* < 0.00001) (Figure 4F). Publication bias could not be excluded for OS, CSS, or PFS by inverted funnel plots. Not publishing negative results was suspected.

### Clinical Significance of NLR in Chemotherapy Patients

#### Correlation of NLR With Oncological Outcomes (Progression-Free Survival, Overall Survival)

**Figure 5:**
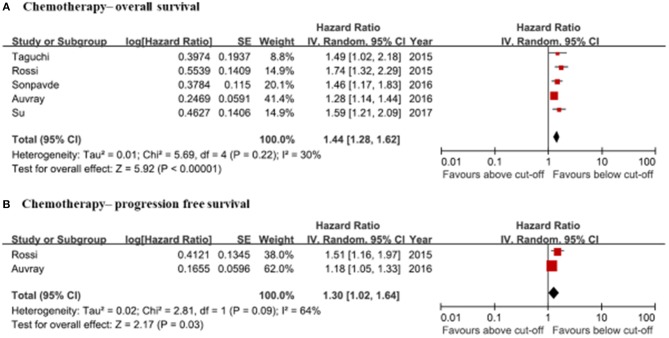
Forrest plots of the relationship between NLRs and clinical information in chemotherapy patients. **(A)** Chemotherapy—overall survival. **(B)** Chemotherapy—progression free survival.

The pooled analysis of OS was based on five studies (29–33). Pretreatment NLRs were significantly associated with OS (HR: 1.44, 95% CI: 1.28–1.62) (Figure 5A). Only two studies reported information on PFS (31, 32). The pooled hazard ratio of PFS in these studies was 1.30 (95% CI: 1.02–1.64) (Figure 5B). Inter-study heterogeneity was moderately present in the meta-analyses of OS (*I*^2^ = 30%, *p* = 0.22) and PFS (*I*^2^ = 64%, *p* = 0.09). Publication bias was not shown by funnel plots. However, the number of studies for PFS was too small to accurately evaluate.

### Clinical Significance of NLR in Nephroureterectomy Patients

#### Correlation of NLR With Oncological Outcomes (Progression-Free Survival, Overall Survival, Cancer-Specific Survival)

**Figure 6:**
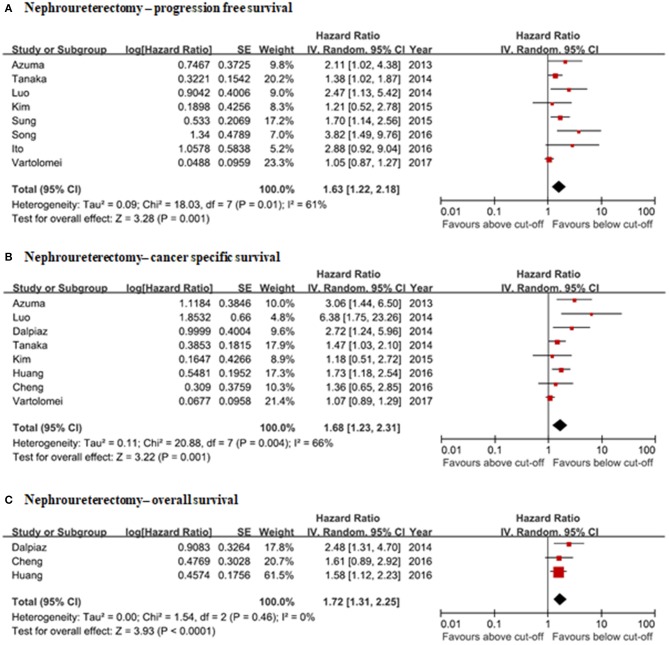
Forrest plots of the relationship between NLRs and clinical information in nephroureterectomy patients. **(A)** Nephroureterectomy—progression free survival. **(B)** Nephroureterectomy—cancer specific survival. **(C)** Nephroureterectomy—overall survival.

Pooled hazard ratios were calculated for PFS, OS, and CSS in this meta-analysis. A total of eight studies reported the relationship between preoperative NLRs and PFS. Preoperative NLRs were significantly associated with PFS (HR: 1.63, 95% CI: 1.22–2.18) (Figure 6A). Preoperative NLRs lower than the cut-off value were also associated with better CSS (HR: 1.68, 95% CI: 1.23–2.31) (Figure 6B). The HR of preoperative NLR for overall survival in nephroureterectomy patients was 1.72 (95% CI: 1.31–2.25) based on the meta-analysis results of three eligible studies (Figure 6C). Heterogeneity among these enrolled studies was moderately present for CSS and PFS but was not present for OS. There was a risk of publication bias with a possible risk of not reporting negative results.

## Discussion

The neutrophil-to-lymphocyte ratio (NLR) is one of the most actively studied biomarkers for predicting disease status in various cancer types (4, 47). The clinical evidence for the usefulness of NLR as a biomarker in urothelial carcinoma has been accumulating over the last several years, but the topic is still under debate. We conducted a systematic review and meta-analysis to assess the correlation between pretreatment NLRs with pathologic features and the prognosis of urothelial carcinoma patients. This study collected a total of 38 studies containing clinical information and oncological outcomes on 16,379 patients. To our knowledge, this is the largest and latest meta-analysis evaluating the clinical significance of pretreatment NLRs for each specific urothelial carcinoma management situation.

Our evidence supports pretreatment NLR as a useful biomarker for assessing disease aggressiveness and oncological outcomes in TURBT, radical cystectomy, chemotherapy, and nephroureterectomy patients. High NLR was associated with muscle invasiveness (OR: 4.27), poor RFS (HR: 2.32), and PFS (HR: 2.45) in TURBT patients. The major focus of trans-urethral management of superficial bladder urothelial carcinoma is controlling recurrence and progression to muscle-invasive disease. If bladder cancer presents with muscle-invasion and a high risk of progression or recurrence, guidelines recommend radical cystectomy (48, 49). The TURBT procedure always has the risk of failing to obtain the detrusor muscle in the specimen, which is important for proper staging and disease management (50). For this reason, repeated TURBTs should be considered in high-risk bladder NMIBC. The pretreatment NLR before TURBT could provide additional information for selecting the proper treatment strategies for the management of bladder urothelial carcinoma. High NLR values were associated with a higher chance of extravesical extension (OR: 1.14), lymph-node positivity (OR: 1.43), and worse oncological outcomes. The pooled hazard ratios for PFS, CSS, and overall survival were 1.2, 1.35, 1.18, respectively. Patients with low pretreatment NLRs showed better response rates after NAC followed by radical cystectomy, with a higher chance of pathologic down-staging (OR: 0.79). The oncological outcomes of chemotherapy and nephroureterectomy also revealed negative correlations with pretreatment NLRs. We presumed that the correlation between high pretreatment NLRs and poor outcomes after treatment for urothelial carcinoma was a result of more aggressive presentations, however, further well-designed studies are needed to clarify this hypothesis.

Although results from many studies favor NLR as a useful biomarker under many clinical situations, its predictive mechanism remains unclear. We generally accepted that there is a correlation between inflammation and cancer, however, a causal relationship is ambiguous. Chronic inflammation caused by infection or toxic materials leads to tumorigenesis in many solid tumors. About 20% of tumors are associated with prior viral, microbial, and parasite infections, including infections with the hepatitis virus, human papillomavirus, *Helicobacter pylori*, and *Schistosoma haematobium* (51). Additionally, recent studies showed an inverse correlation between non-steroid anti-inflammatory drugs (NSAID) and cancer risk (52). Inflammatory cells are recruited to the tumor microenvironment in the situation of tumor advancement with invasion or the distant migration of tumor cells (53). The cancer-related immune response is paradoxical. Molecular evidence has demonstrated that tumor-infiltrated inflammatory cells eradicate nascent tumors (54). However, some studies have shown that increased systemic inflammation enhanced tumor development, progression, and metastasis (55). Because of the unclear mechanism of the NLR to predict disease prognosis, the clinical utility of the NLR is very limited. Moreover, we have only limited evidence for the association of NLR and prognosis compared to important prognostic factors, such as pathologic stage and surgical margins. Thus, a targeted study evaluating the prognostic impact of NLR after adjusting for covariable factors is needed.

The definition of an optimum NLR value is another problem. In studies included in this meta-analysis, the cutoff value of NLR varied from 2.0 to 5.0 and some studies even used NLR as a continuous variable (12, 23, 25). In addition, NLR is a dynamic marker of the systemic immune response. Thus, we could not judge the optimal cutoff value easily. NLR is not only related to bladder cancer but also related to many benign and malignant diseases. Therefore, it can be increased without the advancement of tumors. Recently, some studies used a derived NLR introduced by Proctor et al. (56) that makes the clinical utility of NLR more complex.

This systematic review and meta-analysis had some limitations. First, most studies on the relationship of NLR with many clinical parameters and oncological outcomes were retrospectively designed. There was no prospective, randomized controlled study that met our search criteria. Variable differences in study design, patient numbers, the definition of oncological outcomes, and NLR cutoff values also affected the inter-study heterogeneity. Publication bias was another limitation, especially in the sub-group analysis of cystectomy, and nephroureterectomy patients. In addition, we could not include immune-oncological agent targeted studies, which are promising treatments for advanced urothelial carcinoma and thought to be strongly correlated with systemic immune responses. Finally, this meta-analysis used articles written in English only. Thus, we could not exclude language bias in the favorable positive results. Despite these limitations, this study-level meta-analysis provided a generalized view of NLRs on disease aggressiveness, oncological outcomes, and treatment responses in patients with urothelial carcinoma.

## Conclusion

This study-level meta-analysis showed that pretreatment NLRs were useful biomarkers for disease aggressiveness, oncological outcomes, and treatment responses in the management of urothelial carcinoma. However, inter-study heterogeneity, the possibility of publication bias, the limited number of eligible studies, and no randomized controlled study were limitations of the study. A large, well-designed, prospective study is needed to provide clear evidence that the pretreatment NLR is a useful biomarker for urothelial carcinoma.

## Author Contributions

JK: conception and design. CJ, CK, HK, and JK: collection and assembly of data. JS: manuscript writing. All authors data analysis and interpretation, final approval of manuscript, and accountable for all aspects of the work.

### Conflict of Interest

The authors declare that the research was conducted in the absence of any commercial or financial relationships that could be construed as a potential conflict of interest.

## Supplementary Material

Supplementary Data 1 Funnel plots of each analysis.

Supplementary Data 2 New Castle Ottawa Scale Summary: The review authors' judgments on each parameter for each included study.

Supplementary Data 3 The New Castle Ottawa Scale detailed review of each study.
