# Supplementary material for: Clinical Significance of Pre-treated Neutrophil-Lymphocyte Ratio in the Management of Urothelial Carcinoma: A Systemic Review and Meta-Analysis
Source: Front Oncol. 2019 Dec 16;9:1365. doi: 10.3389/fonc.2019.01365 (PMC6927426; doi:10.3389/fonc.2019.01365)

Supplementary data 1. Funnel plot of each analysis.

A. TURBT - invasiveness

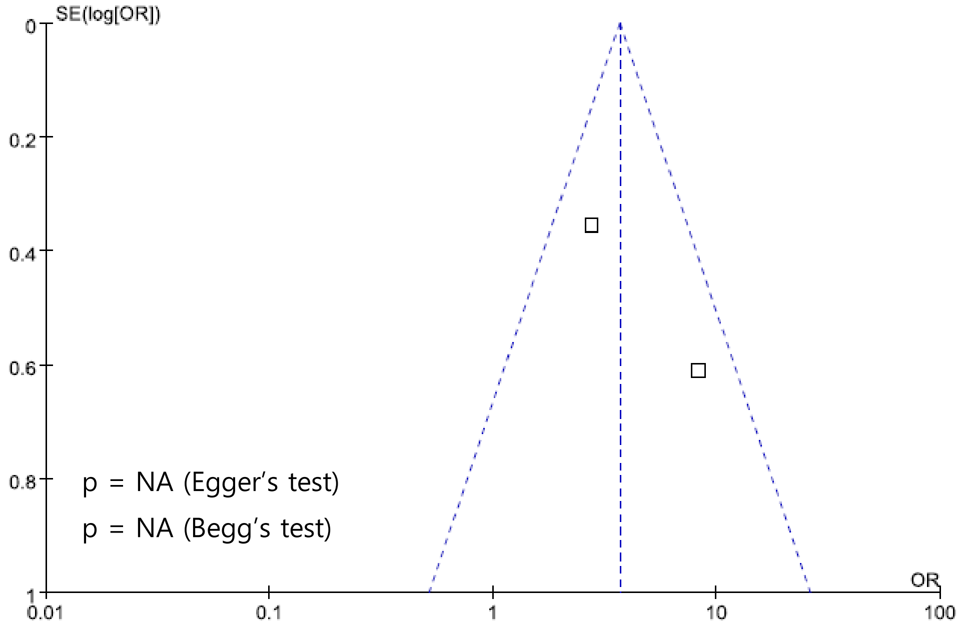

B. TURBT – recurrence free survival

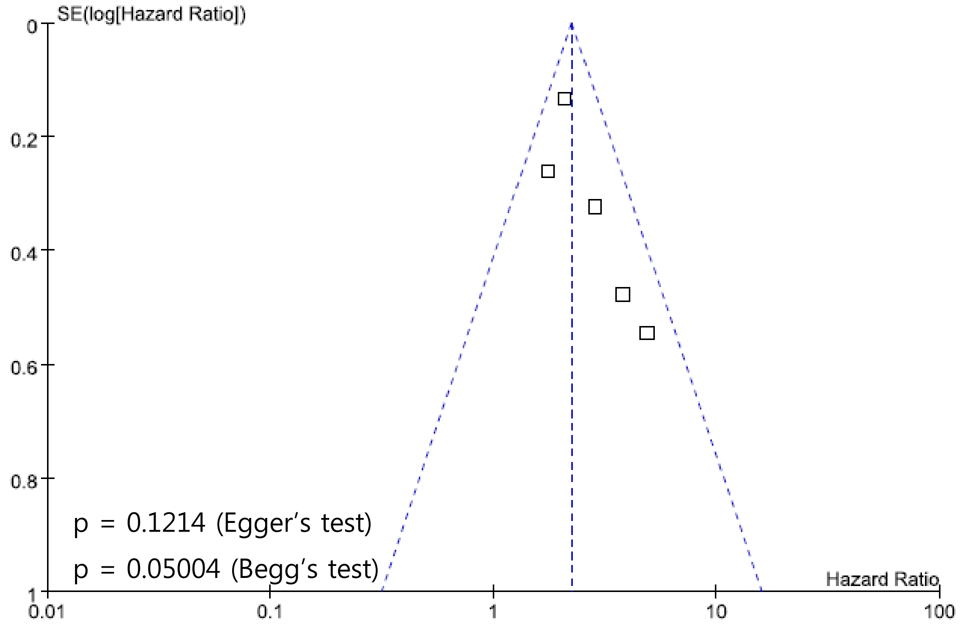

C. TURBT – progression free survival

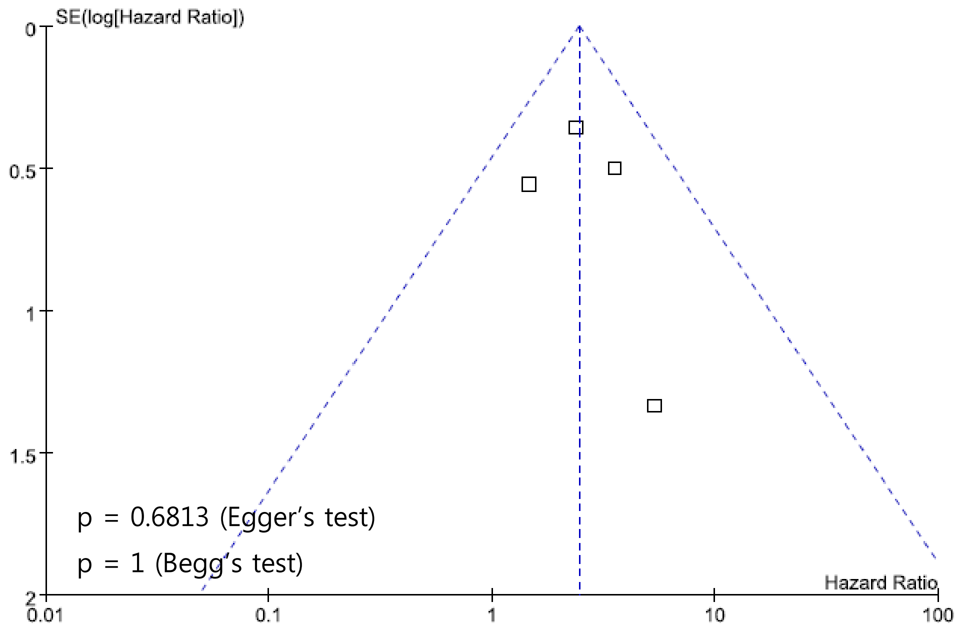

D. Cystectomy – extravesical invasion

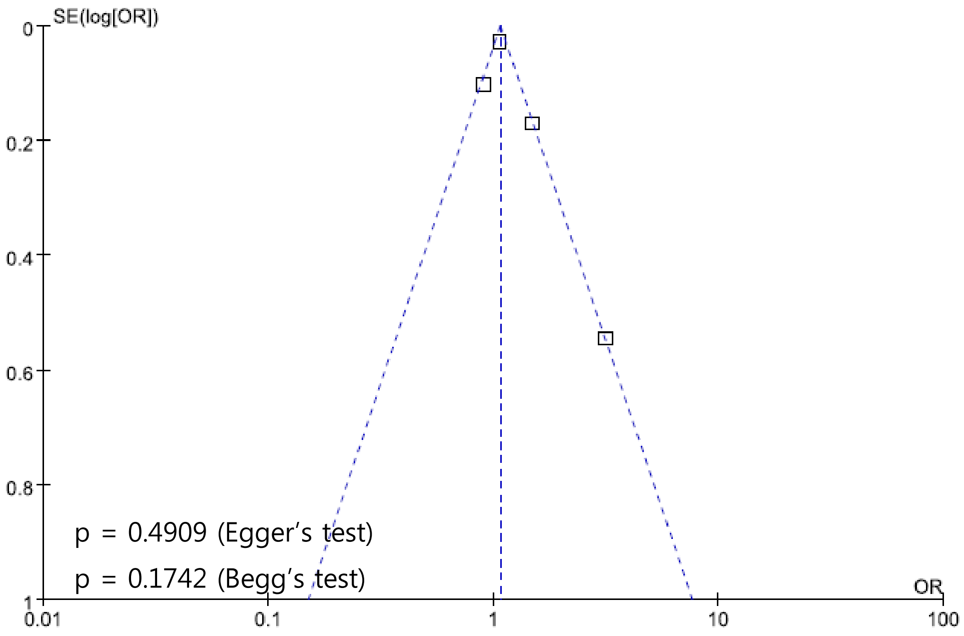

E. Cystectomy – Lymph node positivity

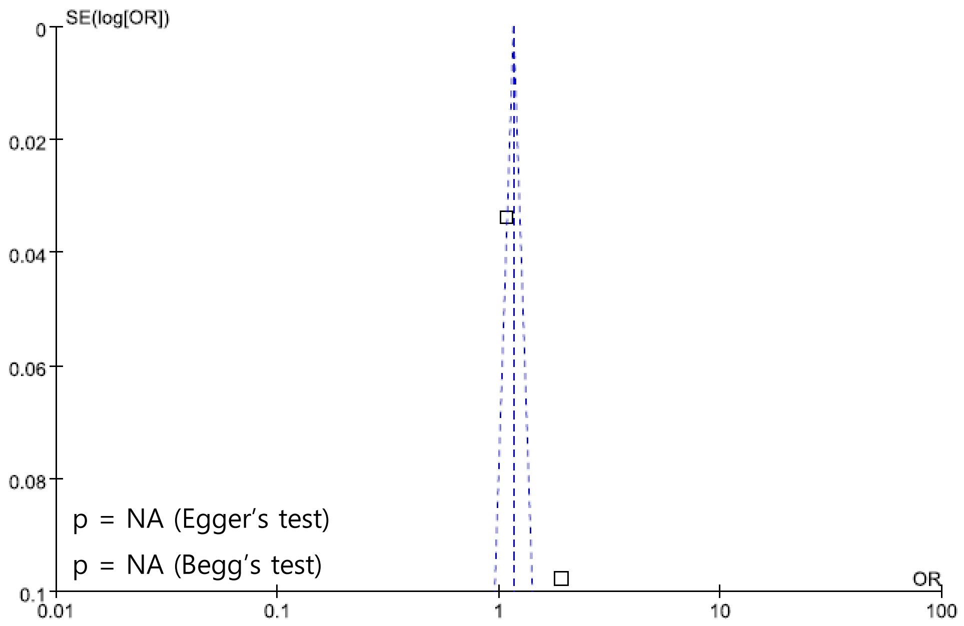

F. Cystectomy – down-staging after neoadjuvant chemotherapy

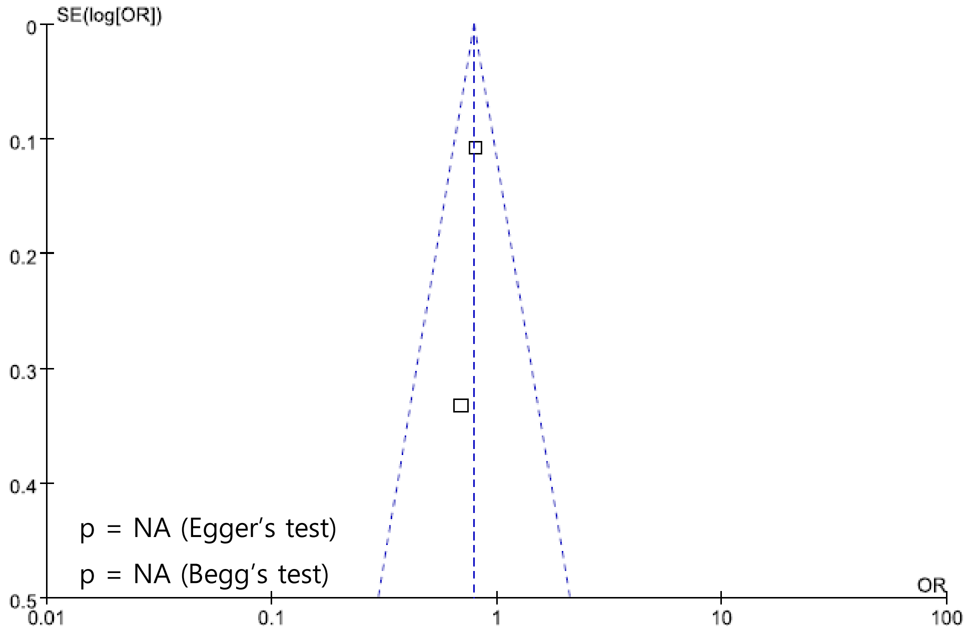

G. Cystectomy- progression free survival

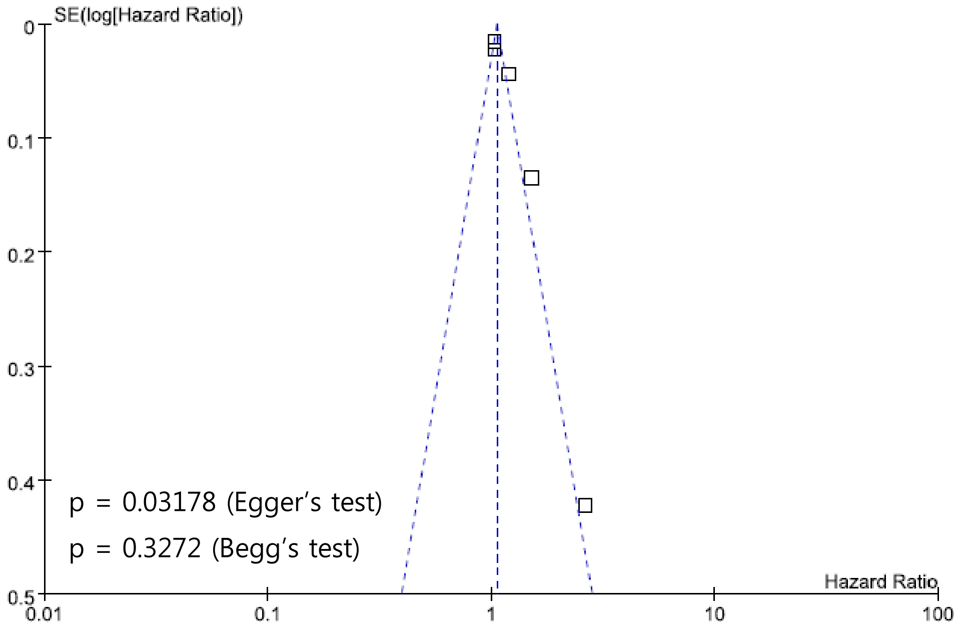

H. Cystectomy – Overall survival

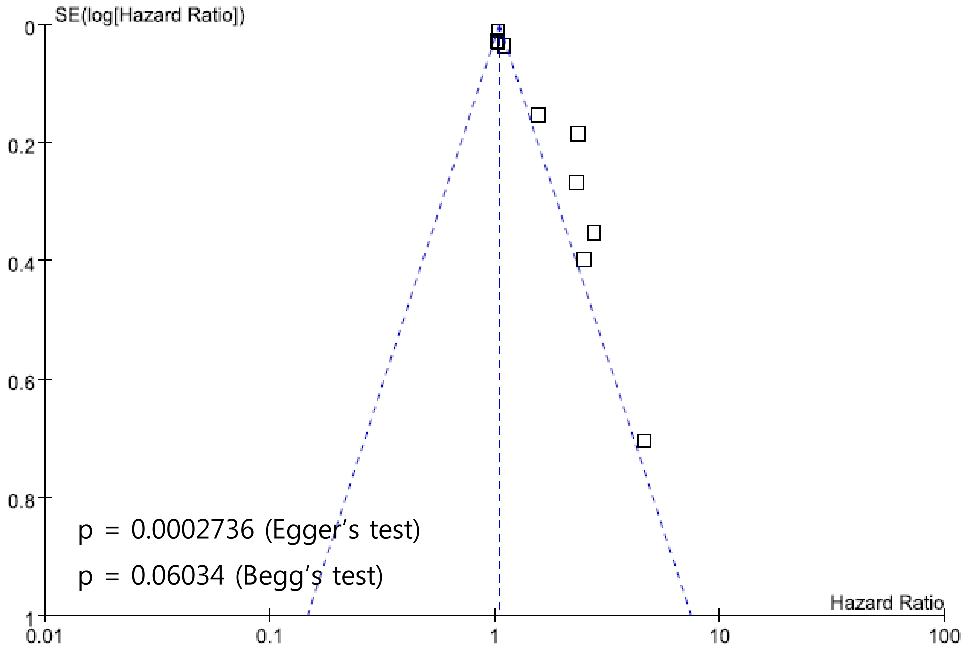

I. Cystectomy- cancer specific survival

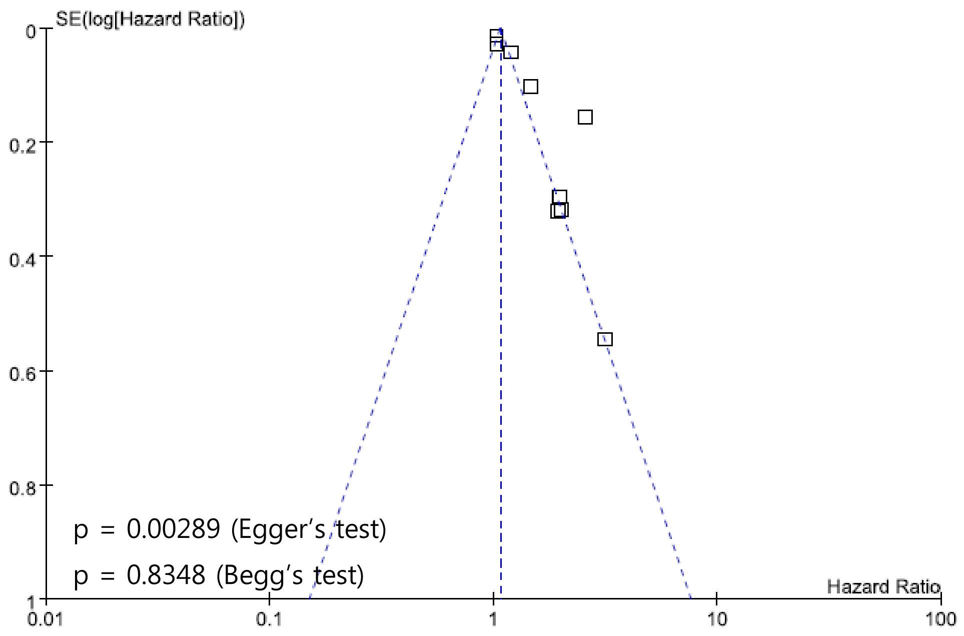

J. Chemotherapy – overall survival

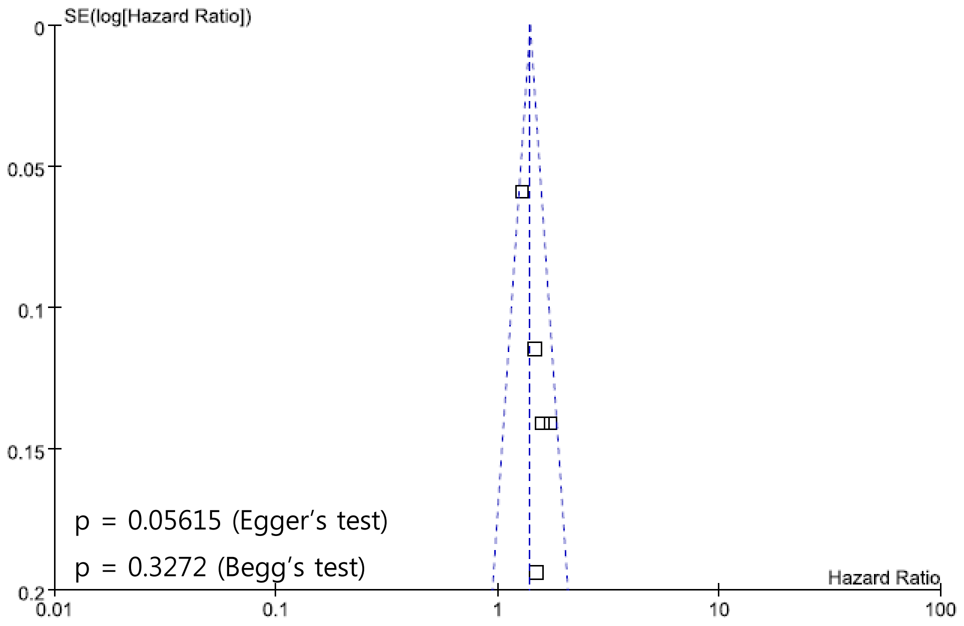

K. Chemotherapy – progression free survival

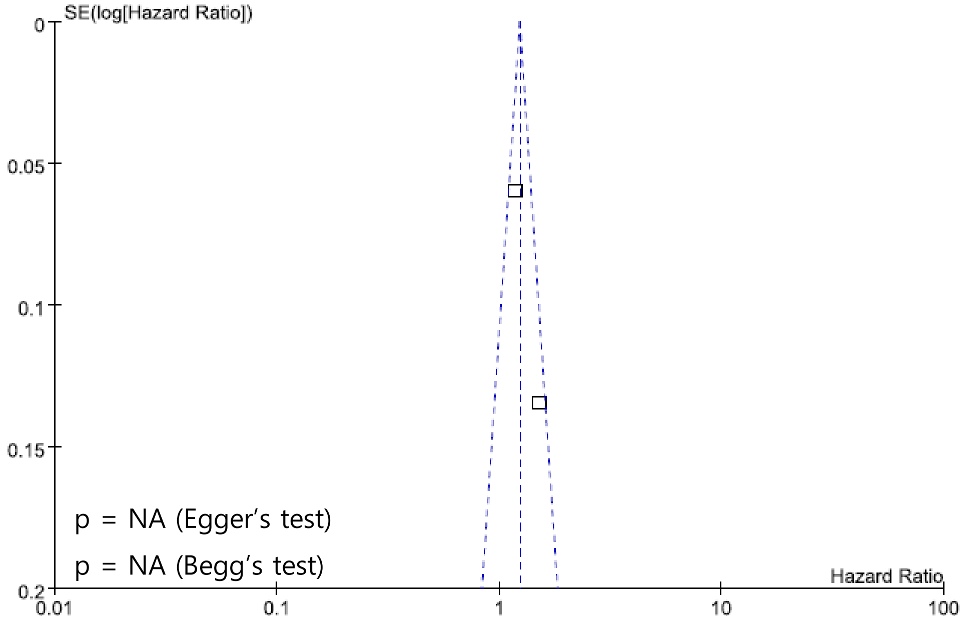

L. Nephroureterectomy – progression free survival

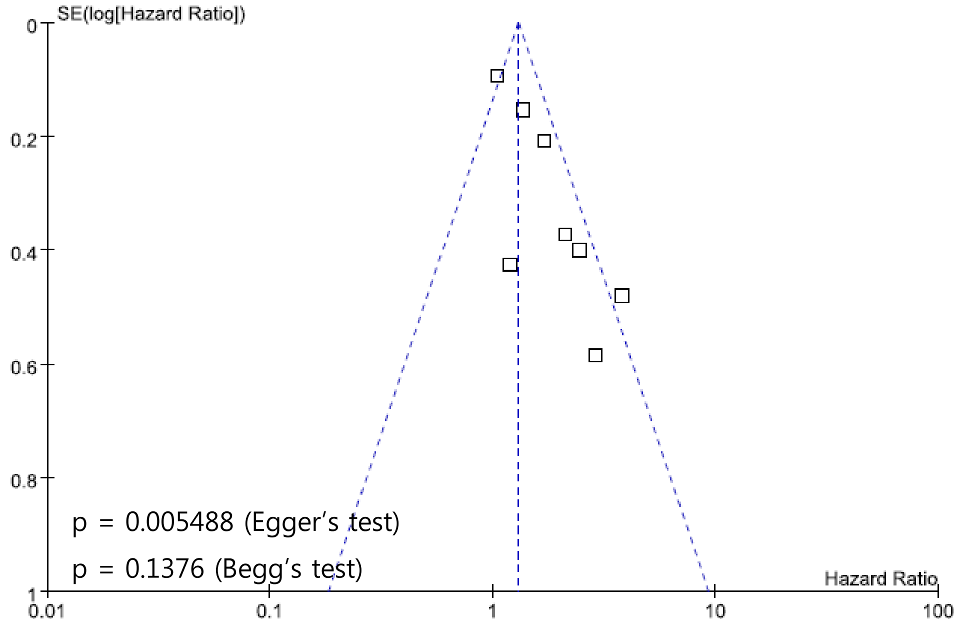

M. Nephroureterectomy – cancer specific survival

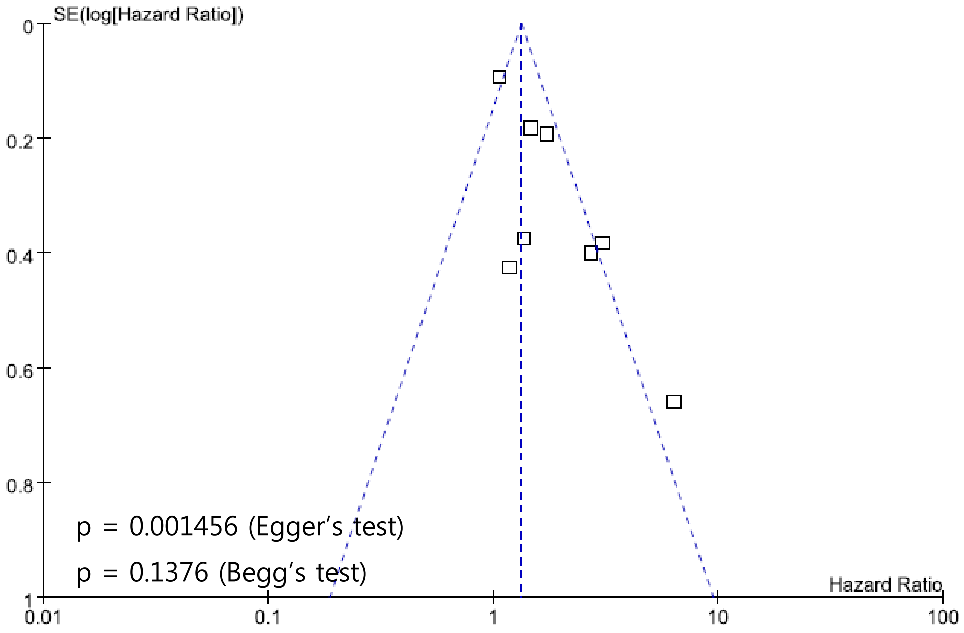

N. Nephroureterectomy – overall survival

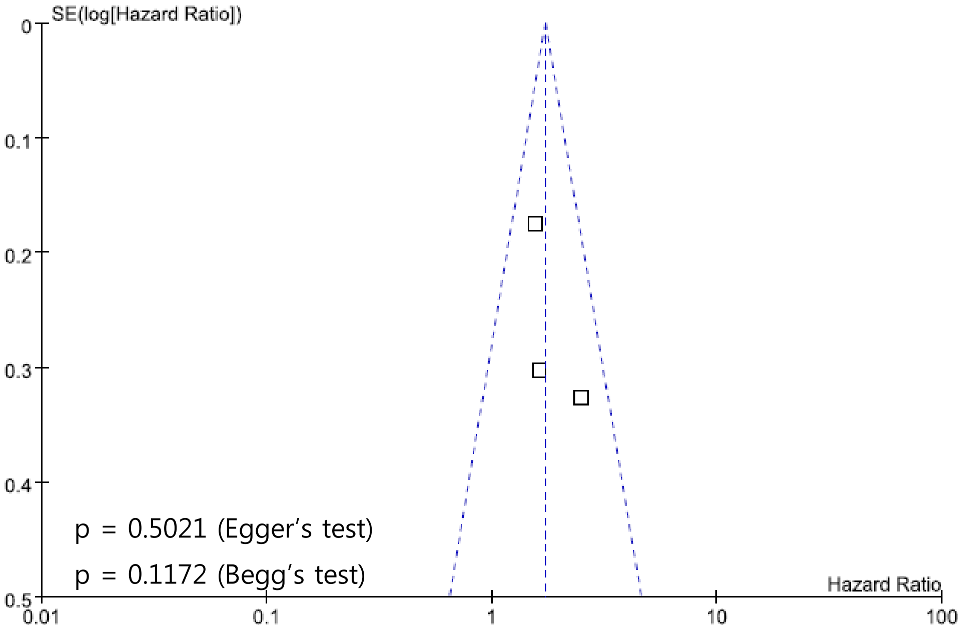

Supplement: Supplementary Data 1 — Funnel plots of each analysis. [file Data_Sheet_1.PDF]
