# Supplementary material for: Clinical Significance of Pre-treated Neutrophil-Lymphocyte Ratio in the Management of Urothelial Carcinoma: A Systemic Review and Meta-Analysis
Source: Front Oncol. 2019 Dec 16;9:1365. doi: 10.3389/fonc.2019.01365 (PMC6927426; doi:10.3389/fonc.2019.01365)
Supplement: Supplementary Data 2 — New Castle Ottawa Scale Summary: The review authors' judgments on each parameter for each included study. [file Data_Sheet_2.PDF]

Supplementary Data 2. New Castle Ottawa Scale Summary: review authors' judgements about each parameter for each included study.

	Representativeness of the exposed cohort	Selection of the non-exposed cohort	Ascertainment of exposure	Demonstration that outcome of interest was not present at start of study	Controls for Most importat factor	Study controls for any additional factor	Assessment of outcome	Was follow-up long enough for outcomes to occur	Adequacy of follow up of cohorts
Auvray 2016	+	+	+	+	+	+	+	+	+
Azuma 2013	+	+	+	+	+	+	+	+	+
Bhindi 2016	+	+	+	+	+	+	+	+	?
Buisan 2017	+	+	+	+	+	+	+	+	+
Camtosun 2017	+	+	+	+	?	?	+	−	?
Can 2012	+	+	+	−	?	?	+	+	+
Cheng 2016	+	+	+	+	+	+	+	+	+
D'Andrea 2017	+	+	+	+	+	+	+	+	+
Dalpiaz 2014	+	+	+	+	+	+	+	+	+
Favilla 2016	+	+	+	+	+	+	+	+	+
Gondo 2012	+	+	+	+	−	+	+	+	+
Huang 2016	+	+	+	+	+	+	+	+	+
Ito 2016	+	+	+	+	+	+	+	?	+
Kawahara 2016	+	+	+	+	+	+	+	−	?
Kim 2015	+	+	+	+	+	+	+	+	+
Krane 2013	+	+	+	+	+	+	+	?	+
Ku 2015	+	+	+	+	+	+	+	+	+
Lee 2015	+	+	+	?	+	?	+	+	+
Luo 2014	+	+	+	+	+	+	+	+	+
Mano 2015	+	+	+	+	+	+	+	−	?
Morizawa 2016	+	+	+	+	+	+	+	+	?
Nguyen 2016	+	+	+	+	+	+	+	?	?
Ogihara 2016	+	+	+	+	+	+	+	+	+
Ojerholm 2017	+	+	+	+	+	?	+	+	+
Ozcan 2015	+	+	+	+	+	+	+	?	−
Ozyalvaci 2015	+	+	+	+	−	+	+	+	−
Potretzke 2014	+	+	+	+	+	+	+	+	+
Rossi 2015	+	+	+	+	+	+	+	+	+
Seah 2015	+	+	+	+	+	+	+	+	+
Song 2016	+	+	+	+	+	+	+	+	+
Sonpavde 2016	+	+	+	+	?	+	+	+	+
Su 2017	+	+	+	+	+	+	+	+	+
Sung 2015	+	+	+	+	+	+	+	+	+
Taguchi 2015	+	+	+	+	+	+	+	+	+
Tanaka 2014	+	+	+	+	+	+	+	+	+
Vartolomei 2017	+	+	+	+	+	+	+	+	+
Viers 2014	+	+	+	+	+	+	+	+	+
Yoshida 2016	+	+	+	+	+	+	+	+	+

+	**Good quality**
?	**Uncertain**
−	**Low quailty**
