# Supplementary material for: Clinical Significance of Pre-treated Neutrophil-Lymphocyte Ratio in the Management of Urothelial Carcinoma: A Systemic Review and Meta-Analysis
Source: Front Oncol. 2019 Dec 16;9:1365. doi: 10.3389/fonc.2019.01365 (PMC6927426; doi:10.3389/fonc.2019.01365)
Supplement: Supplementary Data 3 — The New Castle Ottawa Scale detailed review of each study. [file Data_Sheet_3.PDF]

Studies

Included studies

Camtosun 2017

References

Ahmet Camtosun, Huseyin Celik, Ramazan Altintas, Ibrahim Topcu, Cemal Tasdemir. Does preoperative neutrophil-to-lymphocyte ratio have a value in predicting recurrence in bladder tumour after intravesical BCG treatment? Biomedical Research 2017;28(1):36-40.

Characteristics

**Methods**	Retrospective
**Participants**	TURBR, NMIBC, BCG, 89
**Interventions**	pre-treatment NLR
**Outcomes**	Recurrence
**Notes**	

Risk of bias table

**Bias**	**Authors' judgement**	**Support for judgement**
Representativeness of the exposed cohort	Low risk	Somewhat representative
Selection of the non-exposed cohort	Low risk	Drawn from the same community as the exposed cohort
Ascertainment of exposure	Low risk	Secure record (Laboratory results)
Demonstration that outcome of interest was not present at start of study	Low risk	Yes
Controls for Most importat factor	Unclear risk	Cohorts are not comparable on the basis of the design or analysis controlled for confounders
Study controls for any additional factor	Unclear risk	Cohorts are not comparable on the basis of the design or analysis controlled for confounders
Assessment of outcome	Low risk	Record linkage
Was follow-up long enough for outcomes to occur	High risk	No. 28.7 monts (12-78), too short
Adequacy of follow up of cohorts	Unclear risk	Almost 1/3 show recurrence, no description

Results data

Can 2012

References

C.Can . B.Baseskioglu, M.Yılmaz, E. Colak , A. Ozen, A. Yenilmez. Pretreatment parameters obtained from peripheral blood sample predicts invasiveness of bladder carcinoma. Urologia Internationalis 2012;89(4):468-472. [DOI: 10.1159/000343278]

Characteristics

**Methods**	Retrospective
**Participants**	TURBT, MIBC + NMIBC, 182
**Interventions**	pre-operative NLR (2.57)
**Outcomes**	Muscle invasiveness
**Notes**	

Risk of bias table

**Bias**	**Authors' judgement**	**Support for judgement**
Representativeness of the exposed cohort	Low risk	Somewhat representative
Selection of the non-exposed cohort	Low risk	Drawn from the same community as the exposed cohort
Ascertainment of exposure	Low risk	Secure record (laboratory result)
Demonstration that outcome of interest was not present at start of study	High risk	No. We don't know time sequence relation of NLR to pathologic aggressiveness.
Controls for Most importat factor	Unclear risk	Laboratory result (PLT, NLR), Age, Gender is not only related factor with pathologic aggressieness. Clinical T stage or CIS, urine cytology were more important
Study controls for any additional factor	Unclear risk	not much information.
Assessment of outcome	Low risk	Record linkage
Was follow-up long enough for outcomes to occur	Low risk	Medican follow up was 45 months and range of 6–180 months. But this study not needs long follow up
Adequacy of follow up of cohorts	Low risk	MIBC : NMIBC = 102 : 80

Results data

Favilla 2016

References

Vincenzo Favilla, Tommaso Castelli, Daniele Urzì , Giulio Reale, Salvatore Privitera, Antonio Salici, Giorgio Ivan Russo, Sebastiano Cimino, Giuseppe Morgia. Neutrophil to lymphocyte ratio, a biomarker in non-muscle invasive bladder cancer: A single-institutional longitudinal study. International Braz J Urol 2016;42(4):658-693. [DOI: 10.1590/S1677-5538.IBJU.2015.0243]

Characteristics

**Methods**	Retrospective
**Participants**	TURBT, NMIBC, 178 patients
**Interventions**	pre treatment NLR level (≥3)
**Outcomes**	5 year recurrence free survival, 5 year progression free survival
**Notes**	

Risk of bias table

**Bias**	**Authors' judgement**	**Support for judgement**
Representativeness of the exposed cohort	Low risk	Somewhat representative
Selection of the non-exposed cohort	Low risk	Drawn from the same community as the exposed cohort
Ascertainment of exposure	Low risk	Secure record (Laboratory results)
Demonstration that outcome of interest was not present at start of study	Low risk	Yes
Controls for Most importat factor	Low risk	Yes, pathologic stage concomitant CIS
Study controls for any additional factor	Low risk	Yes, Smoking, number of tumors, Tumor size
Assessment of outcome	Low risk	Record linkage
Was follow-up long enough for outcomes to occur	Low risk	Yes
Adequacy of follow up of cohorts	Low risk	Subjects lost to follow up unlikely to introduce bias-number lost less than or equal to 20% or description of those lost suggested no different from those followed

Results data

Lee 2015

References

Su-Min Lee, Andrew Russell, Giles Hellawell. Predictive value of pretreatment inflammation-based prognostic scores (Neutrophil-to-lymphocyte ratio, platelet-to-lymphocyte ratio, and lymphocyte-to-monocyte ratio) for invasive bladder carcinoma. Korean Journal of Urology 2015;56(11):749-755. [DOI: 10.4111/kju.2015.56.11.749]

Characteristics

**Methods**	Retrospective
**Participants**	TURBT, NMIBC+MIBC, 226
**Interventions**	NLR, LMR, PLR
**Outcomes**	Pathologic invasiveness
**Notes**	

Risk of bias table

**Bias**	**Authors' judgement**	**Support for judgement**
Representativeness of the exposed cohort	Low risk	Somewhat representative
Selection of the non-exposed cohort	Low risk	Drawn from the same community as the exposed cohort
Ascertainment of exposure	Low risk	Secure record (Laboratory results)
Demonstration that outcome of interest was not present at start of study	Unclear risk	NO, Uncertain
Controls for Most importat factor	Low risk	Clinical, Pathologic feature (T stage, grade , number) included
Study controls for any additional factor	Unclear risk	Uncertain
Assessment of outcome	Low risk	Record linkage
Was follow-up long enough for outcomes to occur	Low risk	Yes
Adequacy of follow up of cohorts	Low risk	175 for NMIBC, 51 for MIBC. Not need follow up interval for this cross-sectional study.

Results data

Mano 2015

References

Roy Mano, Jack Baniel, Ohad Shoshany, David Margel, Tomer Bar-On, Ofer Nativ, Jacob Rubinstein, Sarel Halachmi. Neutrophil-to-lymphocyte ratio predicts progression and recurrence of non–muscle-invasive bladder cancer. Urologic Oncology 2015;33(2):67e1-67e7. [DOI: http://dx.doi.org/10.1016/j.urolonc.2014.06.010]

Characteristics

**Methods**	Retrospective
**Participants**	TURBT, NMIBC, 122
**Interventions**	pre-operative NLR (> 2.41)
**Outcomes**	Recurrence, Progression
**Notes**	

Risk of bias table

**Bias**	**Authors' judgement**	**Support for judgement**
Representativeness of the exposed cohort	Low risk	Somewhat representative
Selection of the non-exposed cohort	Low risk	Drawn from the same community as the exposed cohort
Ascertainment of exposure	Low risk	Secure record (Laboratory results)
Demonstration that outcome of interest was not present at start of study	Low risk	Yes
Controls for Most importat factor	Low risk	Yes, T stage ,Size, Multiplicity and CIS
Study controls for any additional factor	Low risk	Yes, Smoking history
Assessment of outcome	Low risk	Record linkage
Was follow-up long enough for outcomes to occur	High risk	No. 3 year recurrence and progrssion is too short for assessing oncological outcomes
Adequacy of follow up of cohorts	Unclear risk	64% was recurred, Unclear risk, Median follow up for recurrence was 40 months.

Results data

Ogihara 2016

References

Ogihara Koichiro, Kikuchi Eiji,Yuge Kazuyuki,Yanai Yoshinori,Matsumoto Kazuhiro,Miyajima Akira,Asakura Hirotaka,Oya Mototsugu. The Preoperative Neutrophil-to-lymphocyte Ratio is a Novel Biomarker for Predicting Worse Clinical Outcomes in Non- muscle Invasive Bladder Cancer Patients with a Previous History of Smoking. Annals of Surgical Oncology 2016;23:1039-1047. [DOI: 10.1245/s10434-016-5578-4]

Characteristics

**Methods**	Retrospective
**Participants**	TURBT, NMIBC, 605 patients
**Interventions**	pre treatment NLR level (≥2.2)
**Outcomes**	5 year recurrence free survival, 5 year progression free survival
**Notes**	

Risk of bias table

Bias	Authors' judgement	Support for judgement
Representativeness of the exposed cohort	Low risk	Somewhat representative
Selection of the non-exposed cohort	Low risk	Drawn from the same community as the exposed cohort
Ascertainment of exposure	Low risk	Secure record
Demonstration that outcome of interest was not present at start of study	Low risk	Yes
Controls for Most importat factor	Low risk	Yes, Include pathologic stage, grade and concomitant CIS
Study controls for any additional factor	Low risk	Yes, Smoking history etc
Assessment of outcome	Low risk	Record linkage
Was follow-up long enough for outcomes to occur	Low risk	Yes
Adequacy of follow up of cohorts	Low risk	5 year RFS was 68.4%

Results data

Ozyalvacli 2015

References

Mehmet Emin Ozyalvacli, Gulzade Ozyalvacli, Ramazan Kocaaslan, Kursat Cecen, Ugur Uyeturk, Eray Kemahlı, Adnan Gucuk. Neutrophil-lymphocyte ratio as a predictor of recurrence and progression in patients with high-grade pT1 bladder cancer. Can Urol Assoc J 2015;9:126-131. [DOI: 10.5489/cuaj.2523]

Characteristics

**Methods**	Retrospective
**Participants**	TURBT, NMIBC, 166
**Interventions**	pre-treatment NLR
**Outcomes**	5 year progression free survival, 5 year recurrence free survival
**Notes**	

Risk of bias table

Bias	Authors' judgement	Support for judgement
Representativeness of the exposed cohort	Low risk	Somewhat representative
Selection of the non-exposed cohort	Low risk	Drawn from the same community as the exposed cohort
Ascertainment of exposure	Low risk	Secure record (laboratory exam)
Demonstration that outcome of interest was not present at start of study	Low risk	Yes
Controls for Most importat factor	High risk	No, Tumor pathology, CIS data not included
Study controls for any additional factor	Low risk	Yes, Smoking, BCG, MMC use information included
Assessment of outcome	Low risk	Record linkage
Was follow-up long enough for outcomes to occur	Low risk	5-year is feasible for assessing outcome for recurrence and progression
Adequacy of follow up of cohorts	High risk	mean follow up 24.2 month

Results data

D'Andrea 2017

References

David D’Andrea, Marco Moschini, Kilian M. Gust,| Mohammad Abufaraj, Mehmet Özsoy, Romain Mathieu, Francesco Soria, Alberto Briganti, Morgan Rouprêt, Pierre I. Karakiewicz, Shahrokh F. Shariat. Lymphocyte-to-monocyte ratio and neutrophil-to-lymphocyte ratio as biomarkers for predicting lymph node metastasis and survival in patients treated with radical cystectomy. Journal of Surgical Oncology 2017;115(4):455-461. [DOI: 10.1002/jso.24521]

Characteristics

**Methods**	Retrospective
**Participants**	MIBC, non-metastatic disease, 4335
**Interventions**	Lymphocyte to monocyte ratio, Neutrophil to lymphocyte ratio
**Outcomes**	Pathologic aggressiveness prediction (Lymph node positivity, Extravesical disease), Survival (overall, cancer specific, recurrence free survival)
**Notes**	

Risk of bias table

Bias	Authors' judgement	Support for judgement
Representativeness of the exposed cohort	Low risk	Somewhat representative
Selection of the non-exposed cohort	Low risk	Drawn from the same community as the exposed cohort
Ascertainment of exposure	Low risk	Secure record (Laboratory data)
Demonstration that outcome of interest was not present at start of study	Low risk	Yes
Controls for Most importat factor	Low risk	Yes, included pathologic grade, stage and CIS
Study controls for any additional factor	Low risk	Yes, LVI
Assessment of outcome	Low risk	Record linkage
Was follow-up long enough for outcomes to occur	Low risk	5 year is enough to assessing RFS, OS and CSS in MIBC patients
Adequacy of follow up of cohorts	Low risk	Subjects lost to follow up unlikely to introduce bias- number lost less than or equal to 20% or description of those lost suggested no different from those followed 42.4 month 33.3% recurr, 46.6% deceased and 27.5% died due to UCC.

Results data

Ojerholm 2017

References

Eric Ojerholm, Andrew Smith, Wei-Ting Hwang, Brian C. Baumann, Kai N. Tucker, Seth P. Lerner, Ronac Mamtani, Ben Boursi, John P. Christodouleas. Neutrophil-to-lymphocyte ratio as a bladder cancer biomarker: Assessing prognostic and predictive value in SWOG 8710. Cancer 2017;123(5):794-801.

Characteristics

**Methods**	Prospectrive cohort
**Participants**	MIBC, Radical cystectomy, 230 for prognostic analysis, 263 for predictive analysis
**Interventions**	pre-treatment NLR
**Outcomes**	Overall survival
**Notes**	SWOG8710

Risk of bias table

Bias	Authors' judgement	Support for judgement
Representativeness of the exposed cohort	Low risk	Somewhat representative
Selection of the non-exposed cohort	Low risk	Drawn from the same community as the exposed cohort
Ascertainment of exposure	Low risk	Secure record
Demonstration that outcome of interest was not present at start of study	Low risk	Yes
Controls for Most importat factor	Low risk	Yes, T stage category
Study controls for any additional factor	Unclear risk	Yes, LVI, Treatment method
Assessment of outcome	Low risk	Record linkage
Was follow-up long enough for outcomes to occur	Low risk	Median follow up 18.6 yearS
Adequacy of follow up of cohorts	Low risk	Subjects lost to follow up unlikely to introduce bias- number lost less than or equal to 20% or description of those lost suggested no different from those followed.

Results data

Yoshida 2016

References

Takashi Yoshida, Hidefumi Kinoshita, Kenji Yoshida, Takao Mishima, Masaaki Yanishi, Yoshihiro Komai, Motohiko Sugi, Takashi Murota, Gen Kawa, Tadashi Matsuda. Perioperative change in neutrophil-lymphocyte ratio predicts the overall survival of patients with bladder cancer undergoing radical cystectomy. Japanese Journal of Clinical Oncology 2016;46(12):1162-1167. [DOI: 10.1093/jjco/hyw129]

Characteristics

**Methods**	Retrospective
**Participants**	NMIBC+MIBC, Radical cystectomy, 323
**Interventions**	pre-treatment NLR, post-treatment NLR
**Outcomes**	5-year overall survival
**Notes**	

Risk of bias table

Bias	Authors' judgement	Support for judgement
Representativeness of the exposed cohort	Low risk	Somewhat representative
Selection of the non-exposed cohort	Low risk	Drawn from the same community as the exposed cohort
Ascertainment of exposure	Low risk	Secure record
Demonstration that outcome of interest was not present at start of study	Low risk	Yes
Controls for Most importat factor	Low risk	Yes, Pathologic T stage and N stage
Study controls for any additional factor	Low risk	Yes, included Tumor grade, LVI, PSM and adj + neo adj chemotherapy
Assessment of outcome	Low risk	Record linkage
Was follow-up long enough for outcomes to occur	Low risk	5 year is enough to assessin OS in MIBC
Adequacy of follow up of cohorts	Low risk	Subjects lost to follow up unlikely to introduce bias- number lost less than or equal to 20% or description of those lost suggested no different from those followed.

Results data

Buisan 2017

References

Oscar Buisan, Anna Orsola, Joan Areal, Albert Font, Mario Oliveira, Roberto Martinez, Luis Ibarz. Low Pretreatment Neutrophil-to-Lymphocyte Ratio Predicts for Good Outcomes in Patients Receiving Neoadjuvant Chemotherapy Before Radical Cystectomy for Muscle Invasive Bladder Cancer.. Clinical Genitourinary Cancer 2017;15(1):145-151. [DOI: 10.1016/j.clgc.2016.05.004]

Characteristics

**Methods**	Retrospective
**Participants**	Neoadjuvant chemotherapy+Radical cystectomy, MIBC, 205 patient
**Interventions**	pre-treatment NLR level (>2.5)
**Outcomes**	Pathologic response (downstaging), 5 year progression free survival, 5 year cancer specific survival, 5 year overall survival,
**Notes**	

Risk of bias table

Bias	Authors' judgement	Support for judgement
Representativeness of the exposed cohort	Low risk	Somewhat representative
Selection of the non-exposed cohort	Low risk	Drawn from the same community as the exposed cohort
Ascertainment of exposure	Low risk	Secure record
Demonstration that outcome of interest was not present at start of study	Low risk	Yes
Controls for Most importat factor	Low risk	Yes, Included pT stage and pN stage
Study controls for any additional factor	Low risk	Yes, extracted LN,
Assessment of outcome	Low risk	Record linkage
Was follow-up long enough for outcomes to occur	Low risk	5 Year is enough to assessing PFS, CSS, OS for MIBC patients
Adequacy of follow up of cohorts	Low risk	31 months, but various follow up period

Results data

Morizawa 2016

References

Yosuke Morizawa, Makito Miyake, Keiji Shimada, Shunta Hori, Yoshihiro Tatsumi, Yasushi Nakai, Satoshi Anai, Nobumichi Tanaka, Noboru Konishi, Kiyohide Fujimoto. Neutrophil-to-lymphocyte ratio as a detection marker of tumor recurrence in patients with muscle-invasive bladder cancer after radical cystectomy. Urologic Oncology 2016;34(6):257.e11-257.e17.

Characteristics

**Methods**	Retrospective
**Participants**	MIBC, Radical cystectomy, 110
**Interventions**	pre-operative NLR (≥ 2.6)
**Outcomes**	5-year Recurrence free survival, 5-year Cancer specific survival, 5-year Overall survival
**Notes**	

Risk of bias table

Bias	Authors' judgement	Support for judgement
Representativeness of the exposed cohort	Low risk	Somewhat representative
Selection of the non-exposed cohort	Low risk	Drawn from the same community as the exposed cohort

Ascertainment of exposure	Low risk	Secure record
Demonstration that outcome of interest was not present at start of study	Low risk	Yes
Controls for Most importat factor	Low risk	Yes, Pathologic T stage
Study controls for any additional factor	Low risk	Yes, Histologic subtype, LN positivity, PSM
Assessment of outcome	Low risk	Record linkage
Was follow-up long enough for outcomes to occur	Low risk	5 Year is enough to assessing PFS, CSS, OS for MIBC patients
Adequacy of follow up of cohorts	Unclear risk	No statement, but median follow-up is too short in high NLR group

Results data

Kawahara 2016

References

Takashi Kawahara, Kazuhiro Furuya, Manami Nakamura, Kentaro Sakamaki, Kimito Osaka, Hiroki Ito, Yusuke Ito, Koji Izumi, Shinji Ohtake, Yasuhide Miyoshi, Kazuhide Makiyama, Noboru Nakaigawa, Takeharu Yamanaka, Hiroshi Miyamoto4, Masahiro Yao, Hiroji Uemura. Neutrophil-to-lymphocyte ratio is a prognostic marker in bladder cancer patients after radical cystectomy. BMC Cancer 2016;16(1):1-8. [DOI: 10.1186/s12885-016-2219-z]

Characteristics

**Methods**	Retrospective
**Participants**	Radical cystectomy, 74 patients
**Interventions**	Pretreatment NLR level (≥ 2.38)
**Outcomes**	Overall survival, Progression free survival, Pathologic infileration of Lymphocyte
**Notes**	

Risk of bias table

**Bias**	**Authors' judgement**	**Support for judgement**
Representativeness of the exposed cohort	Low risk	Somewhat representative
Selection of the non-exposed cohort	Low risk	Drawn from the same community as the exposed cohort
Ascertainment of exposure	Low risk	Secure record (laboratory results)
Demonstration that outcome of interest was not present at start of study	Low risk	Yes
Controls for Most importat factor	Low risk	Yes, Pathologic T and N stage

Study controls for any additional factor	Low risk	Yes, NAC, Neoadjvant therapy,
Assessment of outcome	Low risk	Record linkage
Was follow-up long enough for outcomes to occur	High risk	No, 24.2 and 29.6 months of follow-up was too short to assesing clinical outcome
Adequacy of follow up of cohorts	Unclear risk	No statement

Results data

Bhindi 2016

References

Bimal Bhindi, Thomas Hermanns, Yanliang Wei, Julie Yu, Patrick O Richard, Marian S Wettstein, Arnoud Templeton, Kathy Li, Srikala S Sridhar, Michael A S Jewett, Neil E Fleshner, Alexandre R Zlotta, Girish S Kulkarni. Identification of the best complete blood count-based predictors for bladder cancer outcomes in patients undergoing radical cystectomy. British Journal of Cancer 2016;114(2):207-212. [DOI: 10.1038/bjc.2015.432]

Characteristics

**Methods**	Retrospective
**Participants**	Radical cystectomy, 418 patients
**Interventions**	pre-treatment NLR, and other CBC based biomarkers
**Outcomes**	Recurrence free survival, Cancer specific survival, Overall survival
**Notes**	

Risk of bias table

**Bias**	**Authors' judgement**	**Support for judgement**
Representativeness of the exposed cohort	Low risk	Somewhat representative
Selection of the non-exposed cohort	Low risk	Drawn from the same community as the exposed cohort
Ascertainment of exposure	Low risk	Secure record (laboratory results)
Demonstration that outcome of interest was not present at start of study	Low risk	Yes
Controls for Most importat factor	Low risk	Yes, pathologic T stage, N stage
Study controls for any additional factor	Low risk	Yes, LVI and PSM
Assessment of outcome	Low risk	Record linkage

Was follow-up long enough for outcomes to occur	Low risk	Median follow up was 40 months (14-72)
Adequacy of follow up of cohorts	Unclear risk	Subjects lost to follow up unlikely to introduce bias- number lost less than or equal to 20% or description of those lost suggested no different from those followed

Results data

Ozcan 2015

References

Cihat Ozcan, Onur Telli, Erdem Ozturk, Evren Suer, Mehmet Ilker Gokce, Omer Gulpinar, Derya Oztuna, Sumer Baltaci, Cagatay Gogus. The prognostic significance of preoperative leukocytosis and neutrophil-to-lymphocyte ratio in patients who underwent radical cystectomy for bladder cancer. Journal of the Canadian Urological Association 2015;9(11):E789-E794. [DOI: 10.5489/cuaj.3061]

Characteristics

**Methods**	Retrospective
**Participants**	Radical cystectomy, 286
**Interventions**	Preoperative NLR,
**Outcomes**	Disease specific survival (CSS)
**Notes**	

Risk of bias table

**Bias**	**Authors' judgement**	**Support for judgement**
Representativeness of the exposed cohort	Low risk	Somewhat representative
Selection of the non-exposed cohort	Low risk	Drawn from the same community as the exposed cohort
Ascertainment of exposure	Low risk	Secure record
Demonstration that outcome of interest was not present at start of study	Low risk	Yes
Controls for Most importat factor	Low risk	Yes, included Pathologic stage, N stage
Study controls for any additional factor	Low risk	Yes. Included PSM, tumor grade, CIS
Assessment of outcome	Low risk	Record linkage
Was follow-up long enough for outcomes to occur	Unclear risk	28 months (0–144), Unclear defined follow up period.
Adequacy of follow up of cohorts	High risk	Follow up rate less than 80% and no description of those lost

Results data

Seah 2015

References

Jo-An Seah, Raya Leibowitz-Amit, Eshetu G. Atenafu, Nimira Alimohamed, Jennifer J. Knox, Anthony M. Joshua, Srikala S. Sridhar. Neutrophil-Lymphocyte Ratio and Pathological Response to Neoadjuvant Chemotherapy in Patients with Muscle-Invasive Bladder Cancer. Clinical Genitourinary Cancer 2015;13(4):e229-e233.

Characteristics

**Methods**	Retrospective
**Participants**	Radical cystectomy + Neoadjuvant chemotherapy , 26
**Interventions**	NLR level at the pre, mid, and post of treatment
**Outcomes**	Pathologic Response
**Notes**	

Risk of bias table

**Bias**	**Authors' judgement**	**Support for judgement**
Representativeness of the exposed cohort	Low risk	Somewhat representative
Selection of the non-exposed cohort	Low risk	Drawn from the same community as the exposed cohort
Ascertainment of exposure	Low risk	Secure record (laboratory results)
Demonstration that outcome of interest was not present at start of study	Low risk	Yes
Controls for Most importat factor	Low risk	Yes, pathologic T stage, N stage
Study controls for any additional factor	Low risk	Yes, Histology, Chemotx cycle and interval from NAC to surgery
Assessment of outcome	Low risk	Independent blind assessment
Was follow-up long enough for outcomes to occur	Low risk	Yes, There are no need to follow up period for this study
Adequacy of follow up of cohorts	Low risk	Yes

Results data

Ku 2015

References

Ja Hyun Ku, Myeon Kang, Hyung Suk Kim, Chang Wook Jeong, Cheol Kwak, Hyeon Hoe Kim. The prognostic value of pretreatment of systemic inflammatory responses in patients with urothelial carcinoma undergoing radical cystectomy. British Journal of Cancer 2015;112(3):461-467.

Characteristics

**Methods**	Retrospective
**Participants**	Radical cystectomy, 419
**Interventions**	pretreatment NLR, Systemic inflammatory response
**Outcomes**	5-year disease specific survival, overall survival
**Notes**	

Risk of bias table

**Bias**	**Authors' judgement**	**Support for judgement**
Representativeness of the exposed cohort	Low risk	Somewhat representative
Selection of the non-exposed cohort	Low risk	Drawn from the same community as the exposed cohort
Ascertainment of exposure	Low risk	Secure record (laboratory result)
Demonstration that outcome of interest was not present at start of study	Low risk	Yes
Controls for Most importat factor	Low risk	Yes, Included pathologic T, N stage
Study controls for any additional factor	Low risk	Yes, PSM, LVI
Assessment of outcome	Low risk	Record linkage
Was follow-up long enough for outcomes to occur	Low risk	Yes, 5-years
Adequacy of follow up of cohorts	Low risk	Subjects lost to follow up unlikely to introduce bias- number lost less than or equal to 20% or description of those lost suggested no different from those followed.

Results data

Viers 2014

References

Boyd R. Viers, Stephen A. Boorjian, Igor Frank, Robert F. Tarrell, Prabin Thapa, R. Jeffrey Karnes, R. Houston Thompson, Matthew K. Tollefson. Pretreatment neutrophil-to-lymphocyte ratio is associated with advanced

pathologic tumor stage and increased cancer-specific mortality among patients with urothelial carcinoma of the bladder undergoing radical cystectomy. European Urology 2014;66(6):1157-1164.

Characteristics

**Methods**	Retrospective
**Participants**	Radical cystectomy, without NAC, 899
**Interventions**	pretreatmnet NLR (within 90d before RC) > 2.7
**Outcomes**	5 year cancer specific survival, overall survival, recurrence free survival
**Notes**	

Risk of bias table

Bias	Authors' judgement	Support for judgement
Representativeness of the exposed cohort	Low risk	Somewhat representative
Selection of the non-exposed cohort	Low risk	Drawn from the same community as the exposed cohort
Ascertainment of exposure	Low risk	Secure record
Demonstration that outcome of interest was not present at start of study	Low risk	Yes
Controls for Most importat factor	Low risk	Yes, Pathologic T, N stage
Study controls for any additional factor	Low risk	Yes, Tumor size, Positive margin, ADJ therapy, LN density
Assessment of outcome	Low risk	Record linkage
Was follow-up long enough for outcomes to occur	Low risk	Yes for 5 - year
Adequacy of follow up of cohorts	Low risk	Subjects lost to follow up unlikely to introduce bias- number lost less than or equal to 20% or description of those lost suggested no different from those followed.

Results data

Potretzke 2014

References

Aaron Potretzke, Luke Hillman, Kelvin Wong, Fangfang Shi, Ryan Brower, Stephanie Mai, Jeremy P. Cetnar, Edwin Jason Abel, Tracy M. Downs. NLR is predictive of upstaging at the time of radical cystectomy for patients with urothelial carcinoma of the bladder. Urologic Oncology 2014;32(5):631-636.

Characteristics

**Methods**	Retrospective
**Participants**	Radical cystectomy, 102
**Interventions**	pre-treatment NLR (within 100 days before surgery)
**Outcomes**	Pathologic upstage (Organ confined to non-organ confined)
**Notes**	

Risk of bias table

Bias	Authors' judgement	Support for judgement
Representativeness of the exposed cohort	Low risk	Somewhat representative
Selection of the non-exposed cohort	Low risk	Drawn from the same community as the exposed cohort
Ascertainment of exposure	Low risk	Secure record
Demonstration that outcome of interest was not present at start of study	Low risk	Yes
Controls for Most importat factor	Low risk	Yes, Pre-operative stage, CIs, LVI
Study controls for any additional factor	Low risk	Yes, Grade, CIS
Assessment of outcome	Low risk	Record linkage
Was follow-up long enough for outcomes to occur	Low risk	Yes, There are no need to follow up period for this study
Adequacy of follow up of cohorts	Low risk	Yes

Results data

Krane 2013

References

L. Spencer Krane, Kyle A. Richards, A. Karim Kader, Ronald Davis, K.C. Balaji, Ashok K. Hemal. Preoperative Neutrophil/Lymphocyte Ratio Predicts Overall Survival and Extravesical Disease in Patients Undergoing Radical Cystectomy. Journal of Endourology 2013;27(8):1046-1050.

Characteristics

**Methods**	Retrospective
**Participants**	Radical cystectomy, 68
**Interventions**	pre-treatment NLR≥ 2.5
**Outcomes**	Overall survival, Cancer specific survival

**Notes**	

Risk of bias table

Bias	Authors' judgement	Support for judgement
Representativeness of the exposed cohort	Low risk	Somewhat representative
Selection of the non-exposed cohort	Low risk	Drawn from the same community as the exposed cohort
Ascertainment of exposure	Low risk	Secure record
Demonstration that outcome of interest was not present at start of study	Low risk	Yes
Controls for Most importat factor	Low risk	Yes, , pathologic T and N stage
Study controls for any additional factor	Low risk	Yes, Operation method, DM and other findings
Assessment of outcome	Low risk	Record linkage
Was follow-up long enough for outcomes to occur	Unclear risk	Too short. 25 months, (13–61)
Adequacy of follow up of cohorts	Low risk	Subjects lost to follow up unlikely to introduce bias- number lost less than or equal to 20% or description of those lost suggested no different from those followed

Results data

Gondo 2012

References

Tatsuo Gondo, Jun Nakashima, Yoshio Ohno, Ozu Choichiro, Yutaka Horiguchi, Kazunori Namiki, Kunihiko Yoshioka, Makoto Ohori, Tadashi Hatano, and Masaaki Tachibana. Prognostic value of neutrophil-to-lymphocyte ratio and establishment of novel preoperative risk stratification model in bladder cancer patients treated with radical cystectomy. Urology 2012;79(5):1085-1091. [DOI: 10.1016/j.urology.2011.11.070]

Characteristics

**Methods**	Retrospective
**Participants**	Radical cystectomy, 189
**Interventions**	pre-tretment NLR
**Outcomes**	One, 3 year, 5 year cancer specific survival
**Notes**	

Risk of bias table

Bias	Authors' judgement	Support for judgement
Representativeness of the exposed cohort	Low risk	Somewhat representative
Selection of the non-exposed cohort	Low risk	Drawn from the same community as the exposed cohort
Ascertainment of exposure	Low risk	Secure record
Demonstration that outcome of interest was not present at start of study	Low risk	Yes
Controls for Most importat factor	High risk	No. no pathologic stage
Study controls for any additional factor	Low risk	Clinical stage, Tumor size, Tumor number, Grade
Assessment of outcome	Low risk	Record linkage
Was follow-up long enough for outcomes to occur	Low risk	Yes
Adequacy of follow up of cohorts	Low risk	Subjects lost to follow up unlikely to introduce bias- number lost less than or equal to 20% or description of those lost suggested no different from those followed.

Results data

Nguyen 2016

References

Daniel P. Nguyen, Bashir Al Hussein Al Awamlh, Padraic O'Malley, Farehin Khan, Patrick J. E., Lewicki David M. Golombos, Douglas S. Scherr. Factors Impacting the Occurrence of Local, Distant and Atypical Recurrences after Robot-Assisted Radical Cystectomy: A Detailed Analysis of 310 Patients. Journal of Urology 2016;196(5):1390-1396.

Characteristics

**Methods**	Retrospective
**Participants**	Robot, Radical cystectomy, 310 patients
**Interventions**	pre-operative NLR
**Outcomes**	Recurrence free survival (Local isolated, Distant, Atypical)
**Notes**	

Risk of bias table

Bias	Authors' judgement	Support for judgement
Representativeness of the exposed cohort	Low risk	Somewhat representative
Selection of the non-exposed cohort	Low risk	Drawn from the same community as the exposed cohort
Ascertainment of exposure	Low risk	Secure record
Demonstration that outcome of interest was not present at start of study	Low risk	Yes
Controls for Most importat factor	Low risk	Yes, Pathologic T, N stage
Study controls for any additional factor	Low risk	Yes, LVI, CIS
Assessment of outcome	Low risk	Record linkage
Was follow-up long enough for outcomes to occur	Unclear risk	2-3 year of recurrence free survival? Short follow up periods (Mean follow up 24 month)
Adequacy of follow up of cohorts	Unclear risk	Follow up rate less than 80% and no description of those lost (189 patients analysis)

Results data

Sonpavde 2016

References

Guru Sonpavde, Gregory R. Pond, Jonathan E. Rosenberg, Dean F. Bajorin, Toni K. Choueiri, Andrea Necchi, Giuseppe Di Lorenzo, Joaquim Bellmunt. Improved 5-Factor Prognostic Classification of Patients Receiving Salvage Systemic Therapy for Advanced Urothelial Carcinoma. Journal of Urology 2015;195(OCT):277-282. [DOI: 10.1016/j.juro.2015.07.111]

Characteristics

**Methods**	Retrospective
**Participants**	Salvage systemic therapy, 708 patients
**Interventions**	pre-treatment NLR
**Outcomes**	Overall survival
**Notes**	

Risk of bias table

Bias	Authors' judgement	Support for judgement
Representativeness of the exposed cohort	Low risk	Somewhat representative
Selection of the non-exposed cohort	Low risk	Drawn from the same community as the exposed cohort
Ascertainment of exposure	Low risk	Secure record
Demonstration that outcome of interest was not present at start of study	Low risk	Yes
Controls for Most importat factor	Unclear risk	No clinical stage information.
Study controls for any additional factor	Low risk	ECOG-PS
Assessment of outcome	Low risk	Record linkage
Was follow-up long enough for outcomes to occur	Low risk	Yes
Adequacy of follow up of cohorts	Low risk	Subjects lost to follow up unlikely to introduce bias- number lost less than or equal to 20% or description of those lost suggested no different from those followed.

Results data

Taguchi 2015

References

Satoru Taguchi, Tohru Nakagawa, Akihiko Matsumoto, Yasushi Nagase, Taketo Kawai, Yoshinori Tanaka, Kanae Yoshida, Sachi Yamamoto, Yutaka Enomoto, Yorito Nose, Toshikazu Sato, Akira Ishikawa, Yukari Uemura, Tetsuya Fujimura, Hiroshi Fukuhara, Haruki Kume, Yukio Homma. Pretreatment neutrophil-to-lymphocyte ratio as an independent predictor of survival in patients with metastatic urothelial carcinoma: A multi-institutional study. International Journal of Urology 2015;22(7):638-643. [DOI: 10.1111/iju.12766]

Characteristics

**Methods**	Retrospective
**Participants**	Salvage chemotherapy, 185
**Interventions**	pre-treatment NLR ≥3, ECOG-PS
**Outcomes**	Overall-survival
**Notes**	

Risk of bias table

Bias	Authors' judgement	Support for judgement
Representativeness of the exposed cohort	Low risk	Somewhat representative
Selection of the non-exposed cohort	Low risk	Drawn from the same community as the exposed cohort
Ascertainment of exposure	Low risk	Secure record
Demonstration that outcome of interest was not present at start of study	Low risk	Yes
Controls for Most importat factor	Low risk	Yes, Clinical stage, metastasis infomation
Study controls for any additional factor	Low risk	Yes, Chemotherpay agent, ECOG-PS
Assessment of outcome	Low risk	Yes
Was follow-up long enough for outcomes to occur	Low risk	13.0 months (7.0-25.5)
Adequacy of follow up of cohorts	Low risk	Subjects lost to follow up unlikely to introduce bias- number lost less than or equal to 20% or description of those lost suggested no different from those followed.

Results data

Rossi 2015

References

Lorena Rossi, Matteo Santoni, Simon J. Crabb, Emanuela Scarpi, Luciano Burattini, Caroline Chau, Emanuela Bianchi, Agnese Savini, Salvatore L. Burgio, Alessandro Conti, Vincenza Conteduca, Stefano Cascinu, Ugo De Giorgi. High Neutrophil-to-lymphocyte Ratio Persistent During First-line Chemotherapy Predicts Poor Clinical Outcome in Patients with Advanced Urothelial Cancer. Annals of Surgical Oncology 2015;22(4):1377-1384. [DOI: 10.1245/s10434-014-4097-4]

Characteristics

**Methods**	Retrospective
**Participants**	First-line chemotherapy, unresectable and metastatic Urothelial carcinoma. 292
**Interventions**	pre-treatment NLR ≥ 3, ECOG-PS, Visceral disease, Hb
**Outcomes**	Progression free survival, Overall survival
**Notes**	

Risk of bias table

Bias	Authors' judgement	Support for judgement
Representativeness of the exposed cohort	Low risk	Somewhat representative
Selection of the non-exposed cohort	Low risk	Drawn from the same community as the exposed cohort
Ascertainment of exposure	Low risk	Secure record
Demonstration that outcome of interest was not present at start of study	Low risk	Yes
Controls for Most importat factor	Low risk	Yes, Stage
Study controls for any additional factor	Low risk	Yes, ECOG-PS
Assessment of outcome	Low risk	Record linkage
Was follow-up long enough for outcomes to occur	Low risk	Yes
Adequacy of follow up of cohorts	Low risk	Subjects lost to follow up unlikely to introduce bias- number lost less than or equal to 20% or description of those lost suggested no different from those followed.

Results data

Auvray 2016

References

Marie Auvray, Reza Elaidi, Mustafa Ozguroglu, Sermin Guven, Hélène Gauthier, Stéphane Culine, Armelle Caty, Charlotte Dujardin, Edouard Auclin, Constance Thibaut, Pierre Combe, Eric Tartour, Stéphane Oudard. Prognostic Value of Baseline Neutrophil-to-Lymphocyte Ratio in Metastatic Urothelial Carcinoma Patients Treated With First-line Chemotherapy: A Large Multicenter Study. Clinical Genitourinary Cancer 2017;15(3):e469-e476. [DOI: 10.1016/j.clgc.2016.10.013]

Characteristics

**Methods**	Retrospective
**Participants**	First-line chemotherapy, metastatic urothelial carcinoma, 280
**Interventions**	pre-treatemtn NLR
**Outcomes**	Overall survival, Progression free survival
**Notes**	

Risk of bias table

Bias	Authors' judgement	Support for judgement
Representativeness of the exposed cohort	Low risk	Somewhat representative
Selection of the non-exposed cohort	Low risk	Drawn from the same community as the exposed cohort
Ascertainment of exposure	Low risk	Secure record
Demonstration that outcome of interest was not present at start of study	Low risk	Yes
Controls for Most importat factor	Low risk	T stage, Grade, metastasis lesion
Study controls for any additional factor	Low risk	Histology, previous tx information, Chmeotherpay agent
Assessment of outcome	Low risk	Record linkage
Was follow-up long enough for outcomes to occur	Low risk	Yes
Adequacy of follow up of cohorts	Low risk	Subjects lost to follow up unlikely to introduce bias- number lost less than or equal to 20% or description of those lost suggested no different from those followed.

Results data

Su 2017

References

Yu-Li Su, Meng-Che Hsieh, Po-Hui Chiang, Ming-Tse Sung, Jui Lan, Hao-Lun Luo, Chun-Chieh Huang, Cheng-Hua Huang, Yeh Tang, Kun-Ming Rau. Novel inflammation-based prognostic score for predicting survival in patients with metastatic urothelial carcinoma. PLOS One 2017;12(1):1-12. [DOI: 10.1371/journal.pone.0169657]

Characteristics

**Methods**	Retrospective
**Participants**	Metastatic Urothelial carcinoma, 256
**Interventions**	pre-treatment NLR ≥ 3.0
**Outcomes**	Overall survival
**Notes**	

Risk of bias table

Bias	Authors' judgement	Support for judgement
Representativeness of the exposed cohort	Low risk	Somewhat representative
Selection of the non-exposed cohort	Low risk	Drawn from the same community as the exposed cohort
Ascertainment of exposure	Low risk	Secure record
Demonstration that outcome of interest was not present at start of study	Low risk	Yes
Controls for Most importat factor	Low risk	ECOG-PS,
Study controls for any additional factor	Low risk	Metastastatic site, Chemoagent, Histologic variant
Assessment of outcome	Low risk	Record linkage
Was follow-up long enough for outcomes to occur	Low risk	Yes
Adequacy of follow up of cohorts	Low risk	Complete follow up- all subject accounted for

Results data

Vartolomei 2017

References

Mihai Dorin Vartolomei, Romain Mathieu, Vitaly Margulis, Jose A. Karam, Morgan Rouprêt, Ilaria Lucca, Aurélie Mbeutcha, Christian Seitz, Pierre I. Karakiewicz, Harun Fajkovic, Christopher G. Wood, Alon Z. Weizer, Jay D. Raman, Nathalie Rioux-Leclercq, Andrea Haitel, Karim Bensalah, Michael Rink, Alberto Briganti, Evanguelos Xylinas, Shahrokh F. Shariat. Promising role of preoperative neutrophil-to-lymphocyte ratio in patients treated with radical nephroureterectomy. World Journal of Urology 2017;35(1):121-130. [DOI: 10.1007/s00345-016-1848-9]

Characteristics

**Methods**	Retrospective
**Participants**	Radical Nephroureterectomy, 2477
**Interventions**	pre-tretment NLR $\geq$ 2.7
**Outcomes**	3 year Refurrence free survival, Cancer specific survival
**Notes**	

Risk of bias table

**Bias**	**Authors' judgement**	**Support for judgement**
Representativeness of the exposed cohort	Low risk	Somewhat representative
Selection of the non-exposed cohort	Low risk	Drawn from the same community as the exposed cohort
Ascertainment of exposure	Low risk	Secure record
Demonstration that outcome of interest was not present at start of study	Low risk	Yes
Controls for Most importat factor	Low risk	Yes, T-stage N stage
Study controls for any additional factor	Low risk	Yes, CIS, LVI and antother informations
Assessment of outcome	Low risk	Record linkage
Was follow-up long enough for outcomes to occur	Low risk	median follow up of 40 months (20-76), 3 year RFS OS
Adequacy of follow up of cohorts	Low risk	Subjects lost to follow up unlikely to introduce bias- number lost less than or equal to 20% or description of those lost suggested no different from those followed

Results data

Huang 2016

References

Jiwei Huang, Yichu Yuan, Yanqing Wang, Jin Zhang, Wen Kong, Haige Chen, Yonghui Chen, Yiran Huang. Prognostic value of preoperative plasma fibrinogen level and platelet-to-lymphocyte ratio (F-PLR) in patients with localized upper tract urothelial carcinoma Jiwei. Oncotarget 2017;8(22):36761-36771. [DOI: 10.18632/oncotarget.13611]

Characteristics

**Methods**	Retrospective
**Participants**	T4N0M0, Urothelial carcinoma uppertract
**Interventions**	pre-treatment PLR(>241.2), NLR (>3.22)
**Outcomes**	Cancer specific survival, Overall Survival
**Notes**	

Risk of bias table

Bias	Authors' judgement	Support for judgement
Representativeness of the exposed cohort	Low risk	Somewhat representative
Selection of the non-exposed cohort	Low risk	Drawn from the same community as the exposed cohort
Ascertainment of exposure	Low risk	Secure record
Demonstration that outcome of interest was not present at start of study	Low risk	Yes
Controls for Most importat factor	Low risk	Yes, T stage/ N stage
Study controls for any additional factor	Low risk	Yes, LVI, Multifocality, Adj ChemoTx
Assessment of outcome	Low risk	Record linkage
Was follow-up long enough for outcomes to occur	Low risk	Yes
Adequacy of follow up of cohorts	Low risk	Subjects lost to follow up unlikely to introduce bias- number lost less than or equal to 20% or description of those lost suggested no different from those followed

Results data

Ito 2016

References

KEIICHI ITO, JUNICHI ASAKUMA, KENJI KURODA, KAZUYOSHI TACHI, AKINORI SATO, AKIO HORIGUCHI, KENJI SEGUCHI, TOMOHIKO ASANO. Preoperative risk factors for extraurothelial recurrence in patients with ureteral cancer treated with radical nephroureterectomy. MOLECULAR AND CLINICAL ONCOLOGY 2015;Aug:530-536.

Characteristics

**Methods**	Retrospective
**Participants**	Radical Nephroureterectomy, N0M0, 71
**Interventions**	pre-treatment NLR
**Outcomes**	Progression (1-3 year)
**Notes**	

Risk of bias table

Bias	Authors' judgement	Support for judgement
Representativeness of the exposed cohort	Low risk	Somewhat representative
Selection of the non-exposed cohort	Low risk	Drawn from the same community as the exposed cohort
Ascertainment of exposure	Low risk	Secure record
Demonstration that outcome of interest was not present at start of study	Low risk	Yes
Controls for Most importat factor	Low risk	Yes, T stage
Study controls for any additional factor	Low risk	Tes, Tumor size, Cytology, Age
Assessment of outcome	Low risk	Record linkage
Was follow-up long enough for outcomes to occur	Unclear risk	Uncertain (over 80% was not progressed within follow up period)
Adequacy of follow up of cohorts	Low risk	Subjects lost to follow up unlikely to introduce bias- number lost less than or equal to 20% or description of those lost suggested no different from those followed

Results data

Cheng 2016

References

Yen-Chen Cheng, Chun-Nung Huang, Wen-Jeng Wu, Ching-Chia Li, Hung-Lung Ke, Wei-Ming Li, Hung-Pin Tu, Chien-Feng Li, Lin-Li Chang, Hsin-Chih Yeh. The Prognostic Significance of Inflammation-Associated Blood Cell Markers in Patients with Upper Tract Urothelial Carcinoma. Annals of Surgical Oncology 2016;23(1):343-351. [DOI: 10.1245/s10434-015-4781-z]

Characteristics

**Methods**	Retrospective
**Participants**	Radical Nephroureterectomy, 420
**Interventions**	pre-treatment NLR, WBC, RDW
**Outcomes**	Overall survival, Cancer Specific survival
**Notes**	

Risk of bias table

Bias	Authors' judgement	Support for judgement
Representativeness of the exposed cohort	Low risk	Somewhat representative
Selection of the non-exposed cohort	Low risk	Drawn from the same community as the exposed cohort
Ascertainment of exposure	Low risk	Secure record
Demonstration that outcome of interest was not present at start of study	Low risk	Yes
Controls for Most importat factor	Low risk	Yes, T, N stage
Study controls for any additional factor	Low risk	Yes, OP type, GFR, Tumor location, Grade
Assessment of outcome	Low risk	Record linkage
Was follow-up long enough for outcomes to occur	Low risk	Yes
Adequacy of follow up of cohorts	Low risk	Subjects lost to follow up unlikely to introduce bias- number lost less than or equal to 20% or description of those lost suggested no different from those followed

Results data

Kim 2015

References

Myong Kim, Kyung Chul Moon, Woo Suk Choi, Chang Wook Jeong, Cheol Kwak, Hyeon Hoe Kim, Ja Hyeon Ku. Prognostic value of systemic inflammatory responses in patients with upper urinary tract urothelial carcinoma. 2015 33;10:1439-1457. [DOI: 10.1007/s00345-015-1484-9]

Characteristics

**Methods**	Retrospective
**Participants**	non-metastatic UTUC, NUx, 277
**Interventions**	pre-treatment NLR, derived NLR, inflammatory markers
**Outcomes**	5- year Recurrence free survival, Cancer specific survival
**Notes**	

Risk of bias table

Bias	Authors' judgement	Support for judgement
Representativeness of the exposed cohort	Low risk	Truly representative
Selection of the non-exposed cohort	Low risk	Drawn from the same community as the exposed cohort
Ascertainment of exposure	Low risk	Secure record
Demonstration that outcome of interest was not present at start of study	Low risk	Yes
Controls for Most importat factor	Low risk	Yes, Pathologic stage N stage
Study controls for any additional factor	Low risk	Operation findings, PSM, LVI, CIS
Assessment of outcome	Low risk	Record linkage
Was follow-up long enough for outcomes to occur	Low risk	5- year
Adequacy of follow up of cohorts	Low risk	Subjects lost to follow up unlikely to introduce bias-number lost less than or equal to 20% or description of those lost suggested no different from those followed.

Results data

Sung 2015

References

Hyun Hwan Sung, Hwang Gyun Jeon, Byong Chang Jeong and Seong Il Seo, Seong Soo Jeon, Han-Yong Choi, Hyun Moo Lee. Clinical significance of prognosis using the neutrophil-lymphocyte ratio and erythrocyte sedimentation rate in patients undergoing radical nephroureterectomy for upper urinary tract urothelial carcinoma. BJU International 2015;115(4):587-594. [DOI: 10.1111/bju.12846]

Characteristics

**Methods**	Retrospective
**Participants**	UTUC, Radical Nephroureterectomy, 410
**Interventions**	pre treatment NLR $\geq$2.5
**Outcomes**	5-year Progression free survival
**Notes**	

Risk of bias table

Bias	Authors' judgement	Support for judgement
Representativeness of the exposed cohort	Low risk	Somewhat representative
Selection of the non-exposed cohort	Low risk	Drawn from the same community as the exposed cohort
Ascertainment of exposure	Low risk	Secure record
Demonstration that outcome of interest was not present at start of study	Low risk	Yes,
Controls for Most importat factor	Low risk	Yes, T stage
Study controls for any additional factor	Low risk	Yes, Multifocality, CIS, histologic variants
Assessment of outcome	Low risk	Record linkage
Was follow-up long enough for outcomes to occur	Low risk	Yes, 5 year
Adequacy of follow up of cohorts	Low risk	Subjects lost to follow up unlikely to introduce bias- number lost less than or equal to 20% or description of those lost suggested no different from those followed.

Results data

Tanaka 2014

References

Nobuyuki Tanaka, Eiji Kikuchi, Kent Kanao, Kazuhiro Matsumoto, Suguru Shirotake, Yasumasa Miyazaki, Hiroaki Kobayashi, Gou Kaneko, Masayuki Hagiwara, Hiroki Ide, Jun Obata, Katsura Hoshino, Nozomi Hayakawa, Takeo Kosaka, Satoshi Hara, Masafumi Oyama, Tetsuo Momma, Yosuke Nakajima, Masahiro Jinzaki, Mototsugu Oya. A Multi-Institutional Validation of the Prognostic Value of the Neutrophil-to-Lymphocyte Ratio for Upper Tract Urothelial Carcinoma Treated with Radical NephroureterectomyNobuyuki. Annals of Surgical Oncology 2014;12:4041-4048. [DOI: 10.1245/s10434-014-3830-3]

Characteristics

**Methods**	Retrospective
**Participants**	Radical nephroureterectomy, UTUC 665
**Interventions**	pre-treatment NLR > 3.0
**Outcomes**	5 year Recurrence freee survival, Cancer Free Survvial
**Notes**	

Risk of bias table

Bias	Authors' judgement	Support for judgement
Representativeness of the exposed cohort	Low risk	Somewhat representative
Selection of the non-exposed cohort	Low risk	Drawn from the same community as the exposed cohort
Ascertainment of exposure	Low risk	Secure record
Demonstration that outcome of interest was not present at start of study	Low risk	Yes
Controls for Most importat factor	Low risk	Yes, pathologic T stagem Nstage
Study controls for any additional factor	Low risk	Yes, CIS, ChemoTx
Assessment of outcome	Low risk	Record linkage
Was follow-up long enough for outcomes to occur	Low risk	5-year
Adequacy of follow up of cohorts	Low risk	Subjects lost to follow up unlikely to introduce bias- number lost less than or equal to 20% or description of those lost suggested no different from those followed

Results data

Luo 2014

References

Hao-Lun Luo, Yen-Ta Chen, Yao-Chi Chuang, Yuan-Tso Cheng, Wei-Ching Lee, Chih-Hsiung Kang, Po-Hui Chiang. Subclassification of upper urinary tract urothelial carcinoma by the neutrophil-to-lymphocyte ratio (NLR) improves prediction of oncological outcome. BJU International 2014;113:144-149. [DOI: 10.1111/bju.12582]

Characteristics

**Methods**	Retrospective
**Participants**	UTUC, Radical nephroureterectomy, 234
**Interventions**	pre-treatment NLR (>3)
**Outcomes**	5- year Cancer Specific survival, Progression Free survival
**Notes**	

Risk of bias table

Bias	Authors' judgement	Support for judgement
Representativeness of the exposed cohort	Low risk	Somewhat representative
Selection of the non-exposed cohort	Low risk	Drawn from the same community as the exposed cohort
Ascertainment of exposure	Low risk	Secure record
Demonstration that outcome of interest was not present at start of study	Low risk	Yes
Controls for Most importat factor	Low risk	Yes, Pathologic T stage
Study controls for any additional factor	Low risk	Yes, Tumor grade, Smokin, CIS, histologic variants
Assessment of outcome	Low risk	Record linkage
Was follow-up long enough for outcomes to occur	Low risk	5-year
Adequacy of follow up of cohorts	Low risk	Subjects lost to follow up unlikely to introduce bias- number lost less than or equal to 20% or description of those lost suggested no different from those followed.

Results data

Dalpiaz 2014

References

Orietta Dalpiaz, Georg C. Ehrlich, Sebastian Mannweiler, Jessica M. Martín Hernández, Armin Gerger, Tatjana Stojakovic , Karl Pummer,Richard Zigeuner, Martin Pichler, Georg C. Hutterer. Validation of pretreatment neutrophil-lymphocyte ratio as a prognostic factor in a European cohort of patients with upper tract urothelial carcinoma. BJU International 2014;114:334-339. [DOI: 10.1111/bju.12441]

Characteristics

**Methods**	Retrospective
**Participants**	UTUC, Radical nephroureterectomy, 202
**Interventions**	Pre-treament NLR
**Outcomes**	Cancer Specific survival, Overall Survival
**Notes**	

Risk of bias table

Bias	Authors' judgement	Support for judgement
Representativeness of the exposed cohort	Low risk	Somewhat representative
Selection of the non-exposed cohort	Low risk	Drawn from the same community as the exposed cohort
Ascertainment of exposure	Low risk	Secure record
Demonstration that outcome of interest was not present at start of study	Low risk	Yes
Controls for Most importat factor	Low risk	Yes, pathologic T stage,
Study controls for any additional factor	Low risk	Yes, Tumor grade, LVI, ECOG-PS
Assessment of outcome	Low risk	Record linkage
Was follow-up long enough for outcomes to occur	Low risk	Yes, 5 year
Adequacy of follow up of cohorts	Low risk	Subjects lost to follow up unlikely to introduce bias- number lost less than or equal to 20% or description of those lost suggested no different from those followed

Results data

Azuma 2013

References

Takeshi Azuma, Yukihide Matayoshi, Keiko Odani, Yohsuke Sato, Yujiro Sato, Yasushi Nagase, Masaya Oshi. Preoperative neutrophil-lymphocyte ratio as an independent prognostic marker for patients with upper urinary tract urothelial carcinoma. Clinical Genitourinary Cancer 2013;11(3):337-341. [DOI: 10.1016/j.clgc.2013.04.003]

Characteristics

**Methods**	Retrospective
**Participants**	URUC, radical nephroureterctomy, 137
**Interventions**	pre-treatmen NLR ≥2.5
**Outcomes**	5 year Recurrence free survival, Cancer specific survival
**Notes**	

Risk of bias table

Bias	Authors' judgement	Support for judgement
Representativeness of the exposed cohort	Low risk	Somewhat representative
Selection of the non-exposed cohort	Low risk	Drawn from the same community as the exposed cohort
Ascertainment of exposure	Low risk	Secure record
Demonstration that outcome of interest was not present at start of study	Low risk	Yes
Controls for Most importat factor	Low risk	Yes , pathologic T stage
Study controls for any additional factor	Low risk	Yes, Grade, LVI, Crp level
Assessment of outcome	Low risk	Record linkage
Was follow-up long enough for outcomes to occur	Low risk	5-year
Adequacy of follow up of cohorts	Low risk	Subjects lost to follow up unlikely to introduce bias- number lost less than or equal to 20% or description of those lost suggested no different from those followed

Results data

Song 2016

References

Xin song, Gui-Ming Zhang, Xiao-cheng Ma, Lei Lu,o Bin Li, Dong-Yue Chai, Li-Jiang Sun. Comparison of preoperative neutrophil–lymphocyte, lymphocyte–monocyte, and platelet–lymphocyte ratios in patients with upper urinary tract urothelial carcinoma undergoing radical nephroureterectomy. OncoTargets and Therapy 2016;9:1399-1407. [DOI: 10.2147/OTT.S97520]

Characteristics

**Methods**	Retrospecrive
**Participants**	URUC, Radical nephroureterectomy 140
**Interventions**	pre-treatment NLR , PLR, LMR
**Outcomes**	5-year Progression free survival
**Notes**	

Risk of bias table

Bias	Authors' judgement	Support for judgement
Representativeness of the exposed cohort	Low risk	Somewhat representative
Selection of the non-exposed cohort	Low risk	Drawn from the same community as the exposed cohort (
Ascertainment of exposure	Low risk	Secure record
Demonstration that outcome of interest was not present at start of study	Low risk	Yes
Controls for Most importat factor	Low risk	Yes, Pathologic stage,
Study controls for any additional factor	Low risk	Yes, Smoking, Tumor grade, LVI
Assessment of outcome	Low risk	Record linkage
Was follow-up long enough for outcomes to occur	Low risk	5 year
Adequacy of follow up of cohorts	Low risk	Subjects lost to follow up unlikely to introduce bias- number lost less than or equal to 20% or description of those lost suggested no different from those followed

Results data

Footnotes
